# Key quality traits in rice grains determining fresh wet rice noodles quality: a multivariate statistical methods across varieties

**DOI:** 10.3389/fpls.2025.1685290

**Published:** 2025-10-15

**Authors:** Yufei Li, Ling Liu, Xin Shu, Fan Zhu, Xinpeng Xiang, Chenhao Wu, Bocheng Tang, Yajun Hu, Guanghui Chen, Yue Wang

**Affiliations:** ^1^ Department of Agronomy, College of Agronomy, Hunan Agricultural University, Changsha, China; ^2^ Yuelu Mountain Laboratory of Hunan Province, Hunan Agricultural University, Changsha, China; ^3^ The Key Laboratory of Crop Germplasm Innovation and Resource Utilization of Hunan Province, Hunan Agricultural University, Changsha, China

**Keywords:** flour rice, fresh wet rice noodles, multiple analysis methods, amylose content, gel consistency

## Abstract

Fresh wet rice noodles is one of the delicacies for young and old in East and Southeast Asia. Its main material is rice. However, there are many varieties of rice. Not all rice is suitable for processing into fresh wet rice noodles. In this study, we used 22 rice varieties as raw materials to produce fresh wet rice noodles. Multiple analysis methods, including correlation analysis, principal component analysis, affiliation function analysis, cluster analysis, stepwise regression, and gray correlation analysis, were used to evaluate and classify the quality of rice and fresh wet rice noodles. Identification indices and measurement standards were determined for the evaluation of the quality of the fresh wet rice noodles. The results showed that the 22 rice varieties had large coefficients of variation for rice gel consistency, amylose content, broken noodle rate, and adhesiveness. There were highly significant correlations between all of the individual indices in terms of fresh wet rice noodle quality. Four principal components (composite decision indices) were extracted using principal component analysis, and the cumulative contribution reached 91.442%. In this study, the composite decision index F3 referred to the amylose content and index F4 was the gel consistency. The results of the principal component composite scores and affiliation functions showed that the quality of the rice and fresh wet rice noodles made from Zhuliangyou 4026 and Yuliangyou 22 was excellent. On the basis of the D-value of the combined evaluation of rice quality and fresh wet rice noodle quality, the 22 rice varieties were clustered into four categories at a distance of 0.20. By combining stepwise regression analysis, correlation analysis, and gray correlation analysis, it was determined that amylose content and gel consistency could be used as rice quality indices for evaluating the quality of fresh wet rice noodles. Moreover, the screening conditions for varieties with an amylose content of between 20% and 25% and a gel consistency of less than 40 mm were found to be suitable for fresh wet rice noodle processing. Therefore, multivariate statistical analysis can be an effective means of evaluating flour rice. This study provides a foundation for the standardization, scalability, and industrialization of fresh wet rice noodle production.

## 1 Introduction

Protein–energy malnutrition in elderly people is becoming more prominent in an increasingly aging society (Boirie et al., 2014). Malnutrition increases the risk of age-related chronic diseases in older persons, yet proper nutritional intake can prevent this type of disease (Amarya et al., 2015). Fresh wet rice noodles are a suitable and popular food for older people (Fu, 2007). Fresh wet rice noodles with higher levels of amylose usually induce a lower glycemic response, making them more favorable for health (Tsukasa, 2019). Fresh wet rice noodles are a delicacy enjoyed by people both young and old in East and Southeast Asia. In China, they are a specialty snack with a long history of approximately 2,300 years (Li et al., 2021). They are made using rice as the main raw material after washing, soaking, grinding, sifting, and drying. Among the various types of noodles, fresh wet rice noodles are very popular. They are simple to make, easy to cook, soft and tender, and easily flavored.

There has been huge market demand for fresh wet rice noodles in China for many years. However, the selection and processing of raw materials causes challenges. There are many workshop-type enterprises involved in the production of fresh wet rice noodles and the degree of industrialization is low. Due to the abundance of rice varieties, there are significant differences in the noodle processing characteristics. Not all rice is suitable for processing into fresh wet rice noodles (Wei et al., 2020; Guo et al., 2022; Mou et al., 2022). Therefore, the screening of the flour rice and the construction of a standardized evaluation system are particularly important.

High-quality fresh wet rice flour is characterized by appropriate hardness and adhesiveness, low cooking loss, and a low broken noodle rate (Yan et al., 2023). Twelve *indica rice* varieties were used in a study of the adaptability of fresh wet rice noodle raw material processing. The results showed that the amylose content, amylopectin content, amylose ratio, fat content, attenuation value, final viscosity value, retrogradation value, and whiteness were significantly related to the comprehensive quality of fresh wet rice noodles. The study also provides appropriate ranges for these indicators (Zhang et al., 2021). However, due to the excessive number of indicators in the evaluation system. This is not conducive to the rapid evaluation of the quality of fresh wet rice noodles. Therefore, relevant research has further explored the core evaluation indicators of rice noodles. The research results of Lei et al. showed that the correlation indices affecting the comprehensive quality of the noodles were amylose content and final viscosity. The thresholds in raw rice suitable for processing into fresh wet rice noodles were higher than 26% and 3852 Pa/s, respectively (Lei et al., 2020). The research results of Gao et al. showed that the protein and amylase contents of rice can be used as core indicators for the evaluation of fresh wet rice noodles. Good-quality fresh wet rice noodles could be obtained from raw material with a protein mass fraction of 6.0%-7.0% and an amylose mass fraction of 21.0%-25.0% (Gao et al., 2015). The research results of Zhou et al. Showed that the mass fraction of amylose content showed highly significant positive correlations with the sensory score of the rice noodles. Amylose content can be used as an index to predict the quality of rice noodles, with the amount suitable for processing fresh wet rice noodles ranging from 22.2 to 26.9% (Zhou et al., 2018). But the results reported by Xuan et al. showed that rice varieties with an amylose content of between 20.0 and 25.0% are suitable for rice noodle production (Xuan et al., 2020). Correlation analysis and factor analysis were used in the above screening of rice noodles quality evaluation indicators and the establishment of standards. We found that in these studies, researchers tried to evaluate the quality of fresh wet rice noodles produced by rice quality related indicators. And the amylose content is the core index of rice noodles evaluation. However, the above research results show that the standards for evaluating rice noodles with amylose content are different. In other words, it is controversial to rely on a single analytical method to evaluate the comprehensive quality of rice noodles. This will directly lead to the lack of a standardized evaluation system. This has seriously constrained the industrialization of fresh wet rice noodle processing. Therefore, it is necessary to use multivariate analysis to comprehensively analyze the adaptability of fresh wet rice noodle raw material processing. Using multivariate analysis methods, to identify key rice quality indicators for screening rice for rice noodles, and further establish a standardized evaluation system.

At the present time, multivariate analysis methods are widely used in the evaluation of different crops. The analysis methods mainly include correlation analysis, affiliation function evaluation, principal component analysis, and multiple linear regression. A comprehensive analysis was conducted on the heat tolerance of 35 summer maize varieties during the big horn stage using multivariate analysis methods including correlation analysis, principal component analysis, the fuzzy membership function method, cluster analysis, stepwise regression analysis, and gray correlation analysis. The results showed that the grain yield, kernels per ear, setting rate, ear length, ear diameter, spindle length of tassels with spikelets, and tassel branch length were determined as the identification indices for heat tolerance of summer maize at the V12 stage (Zhu et al., 2022). A comprehensive analysis of the yield and quality of wheat was carried out using the Shannon–Wiener diversity index (H’), cluster analysis, correlation analysis, principal component analysis, stepwise regression, and linear regression. Five quality traits, i.e., testing weight, grain protein content, water abstraction of flour, evaluation value, and extensibility, can be used as comprehensive performance evaluation indices for wheat varieties (Ding et al., 2020). Sixteen cotton varieties were used as materials. Eighteen physiological indices, including root, stem, and leaf water content, net photosynthetic rate, and leaf water potential, were determined at five different PEG6000 concentrations (0, 2.5, 5, 7.5, and 10%) and a comprehensive evaluation of drought tolerance was conducted. The results showed that these 18 physiological indices were transformed into 5–6 independent composite indices through principal component analysis, and 9 typical indices (Fv/Fm, SWC, LWP, Pro, LMDA, RSPC, RMDA, RSOD, and RCAT) were screened using stepwise regression (Zou et al., 2020). The above studies all show that using multivariate statistical analysis for comprehensive evaluation can avoid the one sidedness of single indicator relative value evaluation. In evaluative research, transform numerous indicators into several comprehensive indicators through principal component analysis. Calculate the comprehensive evaluation D value using the fuzzy membership function method based on the principal component score. Judging the quality of a sample based on its D value is a simple, reliable, and widely applicable evaluation method. At the same time, using multiple analysis methods such as stepwise regression analysis and grey relational analysis to screen core indicators can make the screening results more reliable.

Therefore, this study utilized 22 rice varieties widely cultivated in the mid-lower reaches of the Yangtze River as raw materials for producing fresh wet rice noodles. The rice quality (brown rice rate, milled rice rate, amylose content, and gel consistency) and fresh wet rice noodle quality (broken noodle rate, spit pulp value, rice noodle hardness, adhesiveness, elasticity, chewiness, smell, appearance, texture characteristics, and total sensory evaluation score) were determined. Using multivariate analysis methods (correlation analysis, principal component analysis, affiliation function analysis, cluster analysis, stepwise regression, and gray correlation analysis) to comprehensively analyze each variety. The purpose is to (1) clarify the relationship between rice quality and fresh wet rice noodle quality, (2) determine the core indicator of fresh wet rice noodles evaluation from the rice quality indicators, and (3) establish evaluation criteria for core indicators. The findings may then be used for the standardization, scalability, and industrialization of fresh wet rice noodle production.

## 2 Materials and methods

### 2.1 Test materials

The test materials were 22 rice varieties: Tanliangyou 83 (V1), Zhuliangyou 4026 (V2), Lingliangyou 268 (V3), Lingliangyou 942 (V4), Lingliangyou 102 (V5), Liangyouzao 17 (V6), Zhongzao 39 (V7), Yuliangyou 22 (V8), Xinrongyou 123 (V9), Xiangzaoxian 42 (V10), Xiangzaoxian 32 (V11), Xiangzaoxian 24 (V12), Liangyou 347 (V13), Xiangliangyou No.2 (V14), Longliangyou 018 (V15), Liangyou 5836 (V16), C-liangyou 343 (V17), Huailiangyou 608 (V18), Longliangyou 750 (V19), Shenliangyou 5183 (V20), T-liangyou 817 (V21), and Liangyou 336 (V22). All seeds were provided by the Rice Research Institute of Hunan Agricultural University. The selected varieties are all indica rice varieties widely planted in the middle and lower reaches of the Yangtze River basin. All varieties are excellent cultivated species approved by the national or provincial authorities.

### 2.2 Experimental design and methods

This study was conducted at the Hedong Farm Internship Base of Hunan Agricultural University, Yanxi Town, Liuyang City, Hunan Province, China (113°83′46″E, 28°30′93″N). Sow in mid-April. When the rice seedlings are 25 days old, machine transplanting is used. The plot area of each variety was 40 m^2^ and each experiment was replicated three times. In the experiment, 150 kg/hm^2^ of nitrogen fertilizer, 75 kg/hm^2^ of phosphorus fertilizer, and 75 kg/hm^2^ of potash fertilizer were used. Nitrogen fertilizer was applied at the rate of 40% as a base fertilizer, 30% as a tiller fertilizer, and 30% as a spike fertilizer. Phosphorus fertilizer was applied as a base fertilizer. Potassium fertilizer was applied at 50% as a base fertilizer and 50% as a spike fertilizer. All rice varieties are harvested during their physiological maturity period. Three independent biological replicates were harvested from random regions of each variety. The rice was stored for 90 days under constant temperature and humidity conditions for rice quality and fresh wet rice noodles quality.

### 2.3 Measurement items and methods

#### 2.3.1 Rice quality

100 g of rice was weighed and poured into a grain sheller. After dehulling, the residual grain in the sample was determined and the brown rice and the grains were weighed to an accuracy of 0.1g. Brown rice rate (%) = brown rice weight/(sample grain weight – non-shelled grain weight) × 100. Then, 100 g of brown rice was weighed and poured into a rice milling machine. After the rice samples had cooled to room temperature, the milled rice was weighed to an accuracy of 0.1g. Milled rice rate (%) = milled rice weight/brown rice weight × brown rice rate.

The iodine blue colorimetric method was used to determine the amylose content (Tang et al., 2018). The method of determination was as follows: the rice sample was ground to a fine powder. The samples were required to have at least 95% of the fine powder passing through a sieve of 0.15 mm aperture. The sieved sample was mixed well and placed in a wide-mouth jar for 2 days to equilibrate the moisture. Following this, 20 mg of rice powder was accurately weighed and added to a 100 mL volumetric flask. Then, 0.2 mL of anhydrous ethanol was added and the flask was gently shaken to fully disperse the rice powder. Then, 1.5 mL of 1 mol/L NaOH solution was added and the volumetric flask was shaken again to thoroughly mix the rice powder. Subsequently, the sample was immersed in a boiling water bath for 10 minutes, removed, and allowed to cool to room temperature before being diluted to the desired volume using distilled water. The sample was then placed in a boiling water bath, boiled for 10 minutes, and then removed and allowed to cool to room temperature. The volume was then adjusted to the desired level using distilled water. In the next step, 5 mL of the sample solution was transferred to a 50 mL volumetric flask. Then, 1 mL of 1 mol/L sodium acetate solution and 1 mL of 0.2% iodine and potassium iodide solution were added to the flask, and the sample was diluted to 50 mL. After 20 minutes, the absorbance value was measured at 620 nm.

The gel consistency of the rice was measured by following the method described by Cagampang et al (Cagampang et al., 1973). The method of determination was as follows. First, the rice was ground and sifted, and the moisture was balanced. Then, 100mg of rice powder was accurately weighed and added to a round-bottom test tube. Following this, 0.2mL of 0.025% Alkanna tinctoria ethanol solution was added and the sample shaken with a vortex mixer to fully disperse the rice powder. Then, 2.0 mL of 0.200 mol/L KOH solution was added and the test tube was shaken to mix the rice powder thoroughly and evenly. The test tube was covered with a glass ball and placed into a boiling water bath for 8 minutes. The test tube was removed and the glass ball taken out. The sample was left to cool down for 5 minutes, and then the test tube was placed in an ice water bath at 0°C to cool for 20 minutes. The test tube was removed and immediately placed horizontally in a measuring box with markings. After standing for 1h at 25°C, the flow length of the rice gel in the test tube was immediately measured.

#### 2.3.2 Cooking quality of fresh wet rice noodles

The rice samples were soaked in water at 30°C for 3 h, and then ground into pulp according to the rice/water (mass) ratio of 1:1.8, passed through a 0.25 mm sieve, and stirred well. Then, 60 g of rice paste was weighed out and spread flat on a stainless steel disk, steamed in a steamer for 90 s, and then removed. After cooling at room temperature for 15 minutes, the sample was maintained for 2 h at 4°C before being removed. The rice noodles were then cut into strips measuring 8mm wide and 20 cm long and placed in a zip-lock bag for later use.

The broken noodle rate of the fresh wet rice noodles was calculated by following the method described by Luo et al (Luo et al., 2011). The method of determination was as follows: Twenty 20 cm long fresh wet rice noodles were randomly selected. After cooking in 500 mL boiling water for 1 min, the rice noodle sample was removed and rinsed with cold water. The number of rice vermicelli sticks over 10cm (x_1_) was counted, and the broken noodle rate was calculated according to the following formula: Broken noodle rate (%) = (20 – x_1_)/20 × 100.

The spit pulp value of the fresh wet rice noodles was measured by following the method described by Lei et al (Lei et al., 2020). The method of determination was as follows: the moisture content of the rice noodles (ω) was determined and a 20 g rice noodle sample was weighed (m_0_/g). The samples were boiled in 500 mL boiling water for 2 min, and the volume of the soup was fixed at 500 mL. Then, 50 mL of liquid was aspirated into a constant-weight container (m_1_/g), which was then left to dry at 105 ± 2°C to a constant weight (m_2_/g). The spit pulp value was calculated according to the following formula: Spit pulp value (%) = (10 × (m_2_ – m_1_))/(m_0_ × (1 – ω))×100.

#### 2.3.3 Texture determination of fresh wet rice noodles

The textural properties of the fresh wet rice noodles were analyzed using a TA-XT2i texture instrument (Stable Micro System, UK). The prepared rice noodles were cut into strips 5 cm in length and neatly folded once in the center of the mass spectrometer carrier table. The measurement parameters were as follows: the velocity before measurement was 2 mm/s, the velocity during measurement was 1 mm/s, the velocity after measurement was 1 mm/s, the compression ratio was 50%, the time interval between two compressions was 3.0 s, and the trigger force was 5 g. The texture measurement indices included rice noodle hardness, adhesiveness, elasticity, and chewiness. Each test was repeated 6 times (Wu et al., 2025).

#### 2.3.4 Sensory evaluation of fresh wet rice noodles

A sensory evaluation of the fresh wet rice noodles was made by following the method described by Lei et al (Lei et al., 2020). A tasting panel of seven individuals was formed, and the sensory scoring of the rice noodles was carried out according to **Table 1**. Remove the highest and lowest scores and take the average to avoid the influence of individual subjective factors.

**Table 1:** Criteria for sensory evaluation of wet rice noodles.

Level 1 indicators/scores	Secondary indicators/scores	Specific characteristic description: score
Smell/25 points	Rice aroma/25 points	With rice aroma, rich aroma: 22–25 points
		With rice aroma, the aroma is not obvious: 18–21 points
		No rice aroma, but no peculiar smell: 15–17 points
		No rice aroma, and peculiar smell: 0–14 points
Appearance/35 points	Color/10 points	White: 8–10 points
		Normal: 5–7 minutes
		Yellow or gray: 0–4 points
	Gloss/10 points	Obvious gloss: 8–10 points
		Slightly shiny: 5–7 points
		Matte: 0–4 points
	Structure/15 points	The structure is tight, the chopsticks do not easily break the strip, no merging strips, crushing, cracking: 10–15 points
		No broken noodles, parallel strip, crushed powder, a small amount of cracking: 5–9 points
		Crushed powder, noodles easily broken or have a parallel strip, cracking: 0–4 points
Texture characteristics/40 points	Adhesiveness/10 points	Smooth and non-sticky: 8–10 points
		Basic non-sticky: 5–7 points
		Sticky: 0–4 points
	Hardness/10 points	Moderate hardness: 8–10 points
		Slightly hard or soft: 5–7 points
		Very soft or very hard: 0–4 points
	Chewiness/10 points	Chewy: 8–10 points
		Slightly chewy: 5–7 points
		Non-chewy: 0–4 points
	Rice noodle elasticity/10 points	Elasticity: 8–10 points
		Average elasticity: 5–7 points
		Insufficient elasticity: 0–4 points

### 2.4 Data analysis

Microsoft Excel 2021 was used for data organization and DPS 2016 was used for significance of difference testing (Ducan’s new complex polarity method) and gray correlation analysis. SPSS 25.0 was used for the correlation analysis, principal component analysis, affiliation function analysis, cluster analysis, and automatic linear modeling. The specific calculation process is as follows (Shuang et al., 2022; P et al., 2023; Zhang et al., 2024; Wang et al., 2025; Zhang et al., 2025):


(1) $$\text{Principal component composite score}:\text{Y}=\sum_{j=1}^{p}b_{j}y_{j}$$

In the formula, y_j_ is the principal component score. b_j_ is the information contribution of the jth principal component.


(2) $$\begin{array}{c} \text{Affiliation function value}:\text{U}\left(\text{Xj}\right)\\ =\left(\text{Xj}-\text{Xmin}\right)/\left(\text{Xmax-Xmin}\right) \end{array}$$

In the formula, Xj is the jth composite indicator. j = 1, 2, 3, …, n; Xmin and Xmax are the minimum and maximum values of the jth composite indicator; and U (Xj) is the value of the jth composite indicator’s affiliation function.


(3) $$\text{Weight of comprehensive indicator}:{\text{W}}_{j}={\text{R}}_{j}/\sum_{j=1}^{n}R_{j}$$

In the formula, Wj is the weight of the jth composite indicator; and Rj is the contribution of the jth composite indicator.


(4) $$\text{Combined evaluation value}:\text{D}=\sum_{j=1}^{n}\left[\text{U }\left({\text{X}}_{\text{j}}\right){\text{ W}}_{\text{j}}\right]$$

In the formula, D is the combined evaluation value of the different samples.


(5) $$\text{Gray correlation coefficient}:{\text{ξ}}_{\text{ij}}=\frac{{\text{minΔ}}_{ij}+{\text{ρmaxΔ}}_{ij}}{{\text{Δ}}_{ij}+{\text{ρmaxΔ}}_{ij}}$$

In the formula, minΔij and maxΔij denote the minimum and maximum values of all absolute differences; and ρ is the discrimination coefficient.


(6) $$\text{Gray correlation}:{\text{r}}_{i}=\frac{1}{\text{N}}\sum_{j=1}^{N}{\text{ξ}}_{ij}$$

In the formula, N denotes the number of evaluation indicators.

## 3 Results

### 3.1 Basic characteristics of rice and fresh wet rice noodle quality

#### 3.1.1 Rice quality

There are differences in rice quality among 22 rice varieties (**Table 2**). Among them, the brown rice rate and milled rice rate of C-Liangyou 343 are the highest among the 22 varieties, at 82.07% and 70.99%, respectively. At the same time, the Liangyouzao 17 were the lowest, at 70.87% and 47.07%, respectively. The amylose content of Liangyou 336 showed no significant difference compared to Zhongzao 39, Liangyou 347, Xiangliangyou No.2, Tanliangyou 83, T-you 817, Longliangyou 018, Liangliangyou 942, and Liangyouzao 17, but was significantly higher than other varieties by 45.23% -265.92%. The gel consistency of Lingliangyou 268 is not significantly different from Lingliangyou 102, Liangyouzao 17, Liangyou336, Xiangzaoxian 42, Huailiangyou 608, Xinrongyou 123, Liangyou 347, and Lingliangyou 942, but significantly higher than other varieties by 37.36% -251.43%.

**Table 2:** Rice quality and fresh wet rice noodle quality of the 22 tested varieties.

Varieties	Brown rice rateBrown rice rate (%)	Milled rice rateBrown rice rate (%)	Amylose contentBrown rice rate (%)	Gel consistency (mm)	Broken noodles rate (%)	Spit pulp value(%)	Rice-noodles hardness(g)	Adhesiveness (g·s)	Rice-noodles elasticity	Rice- noodles chewiness	Rice-noodles smell	Rice-noodles appearance	Texture characteristics	Total score of sensory evaluation
Tanliangyou 83	78.89 ± 0.18bc	63.97 ± 0.61fghi	27.17 ± 4.72abcd	50.33 ± 5.33cdef	11.67 ± 1.67ghij	7.23 ± 0.17k	4853.58 ± 42.26h	512.79 ± 7.05g	0.62 ± 0.01gh	2648.63 ± 27.56h	16.6 ± 0.24c	25.8 ± 0.2bc	27.2 ± 0.37d	69.6 ± 0.51e
Zhuliangyou 4026	78.33 ± 0.74bc	65.63 ± 0.26efgh	20.44 ± 1.07bcdef	25.67 ± 0.88gh	3.33 ± 1.67l	5.26 ± 0.11l	6918.38 ± 43.57b	79.63 ± 1.01o	0.81 ± 0a	4325.67 ± 14.04a	18.8 ± 0.2a	28.6 ± 0.24a	30.8 ± 0.2ab	78.2 ± 0.2a
Lingliangyou 268	77.91 ± 1.78bc	61.49 ± 1.82i	9.56 ± 3.07g	79.67 ± 2.96a	18.33 ± 1.67def	16.5 ± 0.26c	3714.75 ± 26.36n	657.36 ± 10.43b	0.6 ± 0.01hi	1455.42 ± 87.3l	13.6 ± 0.4i	19.8 ± 0.37h	20.8 ± 0.2j	54.2 ± 0.58j
Liangliangyou 942	79.7 ± 0.31abc	65.01 ± 0.7efgh	25.9 ± 1.63abcd	61.67 ± 8.82abcd	15 ± 0efgh	14.29 ± 0.24e	4347.02 ± 54.36j	476.82 ± 5.34h	0.59 ± 0i	2253.72 ± 23.61j	13.8 ± 0.37i	21.2 ± 0.2g	24.2 ± 0.37gh	59.2 ± 0.2i
Lingliangyou 102	77.09 ± 0.23c	63.08 ± 0.28hi	20.85 ± 0.49bcde	75 ± 4.16ab	16.67 ± 1.67defg	15.43 ± 0.17d	4527.19 ± 31.49i	534.18 ± 4.48f	0.63 ± 0.01fg	2395.55 ± 13.35i	14.2 ± 0.37hi	23.8 ± 0.37ef	25.4 ± 0.4ef	63.4 ± 0.75h
Liangyouzao 17	70.87 ± 0.29e	47.07 ± 1.18j	25.55 ± 1.13abcd	68.33 ± 12.77abc	11.67 ± 1.67ghij	12.57 ± 0.17g	4151.73 ± 9.08k	605.73 ± 3.72d	0.64 ± 0efg	1017.38 ± 9.25o	15.6 ± 0.24def	25.8 ± 0.2bc	25.6 ± 0.4ef	67 ± 0.55f
Zhongzao 39	78.55 ± 0.7bc	63.26 ± 1.06ghi	29.69 ± 2.12ab	43.33 ± 7.26defg	10 ± 2.89hijk	9.16 ± 0.12ij	5416.18 ± 15.29e	357.99 ± 1.34j	0.64 ± 0.01efg	3258.91 ± 19.48f	16.4 ± 0.24cd	25.2 ± 0.2bcd	26.2 ± 0.2e	67.8 ± 0.49f
Yuliangyou 22	78.09 ± 0.28bc	64.16 ± 0.24fghi	22.25 ± 1.21bcde	33 ± 2fgh	5 ± 0kl	3.19 ± 0.06m	7163.87 ± 27.27a	101.35 ± 1.51n	0.79 ± 0a	3978.25 ± 29.37b	18.8 ± 0.37a	29.2 ± 0.2a	31.2 ± 0.37a	79.2 ± 0.8a
Xinrongyou 123	79.61 ± 0.04abc	66.15 ± 0.44defgh	18.77 ± 1.25defg	66.33 ± 10.73abc	20 ± 2.89de	15.8 ± 0.48d	3826.93 ± 25.98m	587.29 ± 2.01e	0.58 ± 0.01i	1436.72 ± 17.37l	12.8 ± 0.37j	21.2 ± 0.2g	19.8 ± 0.37k	53.8 ± 0.73j
Xiangzaoxian 42	79.15 ± 0.23abc	67.31 ± 0.78bcdef	10.87 ± 2.33fg	68 ± 10.12abc	13.33 ± 1.67fghi	13.47 ± 0.2f	5063.61 ± 40.64g	515.63 ± 2.21fg	0.63 ± 0.01fg	2954.61 ± 23.34g	14.8 ± 0.2fgh	25 ± 0.45cd	25.6 ± 0.51ef	65.4 ± 0.4g
Xiangzaoxian 32	78.38 ± 0.42bc	66.49 ± 0.08cdefg	24.1 ± 2.48bcd	36.33 ± 3.76efgh	10 ± 0hijk	8.64 ± 0.17j	6483.39 ± 23.82c	265.78 ± 2.65k	0.69 ± 0.01bc	3616.51 ± 12.75c	17.6 ± 0.24b	28.4 ± 0.51a	28.6 ± 0.24c	74.6 ± 0.81b
Xiangzaoxian 24	79.53 ± 0.15abc	65.85 ± 0.07efgh	23.06 ± 2.8bcde	22.67 ± 0.88h	8.33 ± 1.67ijkl	7.47 ± 0.11k	5139.57 ± 48.18g	127.16 ± 1.16m	0.66 ± 0.01de	3476.47 ± 13.61d	18 ± 0.32b	28.4 ± 0.4a	28.6 ± 0.4c	74.2 ± 0.97b
Liangyou 347	74.03 ± 1.76d	61.59 ± 2.42i	29.58 ± 2.7ab	65.67 ± 2.91abc	13.33 ± 1.67fghi	8.65 ± 0.11j	5290.88 ± 14.68f	126.43 ± 0.33m	0.68 ± 0cd	3333.16 ± 18.53ef	16.6 ± 0.24c	24.6 ± 0.4de	30 ± 0.32b	71.2 ± 0.2d
Xiangliangyou No.2	77.9 ± 1.9bc	67.35 ± 0.76bcdef	29.27 ± 3.25abc	58 ± 6.56bcd	15 ± 0efgh	9.31 ± 0.11i	5353.58 ± 13.22ef	227.43 ± 5.47l	0.65 ± 0.03ef	2920.49 ± 22.47g	17.8 ± 0.2b	21.2 ± 0.2g	29 ± 0c	68 ± 0.32f
Longliangyou 018	78.41 ± 1.02bc	67.57 ± 0.83bcde	26.1 ± 3.37abcd	45.33 ± 7.75defg	36.67 ± 1.67b	20.04 ± 0.17a	2867.7 ± 17.84q	456.27 ± 5.06i	0.49 ± 0l	1151.18 ± 15.86n	15 ± 0fgh	17 ± 0.32i	23.2 ± 0.2i	55.2 ± 0.2j
Liangyou 5836	77.5 ± 2.32bc	67.15 ± 2.46cdef	22.12 ± 5.08bcde	29.67 ± 2.73gh	6.67 ± 1.67jkl	7.32 ± 0.07k	6130.4 ± 22.73d	127.46 ± 5.13m	0.7 ± 0.01b	3389.42 ± 27.01de	17.4 ± 0.24b	29 ± 0.32a	26.2 ± 0.2e	72.6 ± 0.24c
C-liangyou 343	82.07 ± 0.33a	70.99 ± 0.52a	18.59 ± 1.51defg	55.33 ± 7.17bcde	20 ± 2.89de	14.27 ± 0.16e	4587.27 ± 14.37i	514.43 ± 14.21g	0.52 ± 0k	2043.21 ± 16.82k	14.4 ± 0.24ghi	19.6 ± 0.24h	24.2 ± 0.2gh	58.2 ± 0.2i
Huailiangyou 608	79.34 ± 0.18abc	69.55 ± 0.31abc	23.8 ± 7.42bcd	67.67 ± 3.53abc	21.67 ± 1.67cd	11.56 ± 0.11h	4148.79 ± 17.59k	534.14 ± 3.04f	0.53 ± 0.01k	1334.7 ± 36.5m	13.8 ± 0.2i	26 ± 0.32b	25 ± 0.32fg	64.8 ± 0.2gh
Longliangyou 750	78.66 ± 0.29bc	68.17 ± 0.94abcde	19.15 ± 3.95cdefg	37.33 ± 4.48efgh	41.67 ± 1.67a	18.44 ± 0.05b	3053.86 ± 29.67p	686.68 ± 7.05a	0.54 ± 0jk	885.8 ± 5.46p	13.6 ± 0.24i	15.2 ± 0.37j	18 ± 0l	46.8 ± 0.2k
Shenliangyou 5183	80.45 ± 0.76ab	70.52 ± 0.25ab	13.32 ± 2.35efg	37 ± 6efgh	26.67 ± 1.67c	14.4 ± 0.23e	4014.05 ± 14.38l	465.49 ± 7.77hi	0.55 ± 0j	2182.47 ± 12.92j	16 ± 0cde	19.6 ± 0.24h	23.8 ± 0.2hi	59.4 ± 0.24i
T-you 817	78.55 ± 0.6bc	64.96 ± 0.14efgh	26.46 ± 1.72abcd	52 ± 4.73cdef	26.67 ± 1.67c	13.55 ± 0.1f	4337 ± 56.45j	476.65 ± 8.89h	0.63 ± 0.01fg	1455.13 ± 88.31l	15.2 ± 0.2fg	23.4 ± 0.4f	24.8 ± 0.2fg	63.4 ± 0.24h
Liangyou 336	80.65 ± 0.06ab	69.41 ± 0.21abcd	35 ± 0.74a	68.33 ± 1.45abc	43.33 ± 1.67a	18.89 ± 0.25b	3263.62 ± 21.86o	636.47 ± 12.05c	0.58 ± 0.01i	1134.02 ± 24.62n	16.4 ± 0.24cd	19.6 ± 0.24h	19.2 ± 0.2k	55.2 ± 0.2j

Different lowercase letters after data in the same column indicate significant differences at 0.05 level.

#### 3.1.2 Fresh wet rice noodles quality

There were also differences in fresh wet rice noodles among 22 rice varieties (**Table 2**). Among them, the broken noodles rate of Liangyou 336 is the highest, with no significant difference from Longliangyou 750, followed by Longliangyou 018. It is significantly higher than other varieties by 18.19% -1201.20%. And the spit pulp value of Longliangyou 018 was the highest, significantly higher than the other 21 varieties, reaching 6.09% -528.21%. The rice-noodles hardness of Yuliangyou 22 is the highest, significantly higher than the other 21 varieties, reaching 3.55% -149.81%. The adhesiveness of Longliangyou 750 is the highest, significantly higher than the other 21 varieties, reaching 4.46% -762.34%. Rice-noodles elasticity, chewiness, smell, texture characteristics, and Total score of sensory evaluation are all ranked high in Zhuliangyou 4026 and Yuliangyou 22. The rice-noodles appearance of Yuliangyou 22, Liangyou 5836, Zhuliangyou 4026, Xiangzaoxian 24, and Xiangzaoxian 32 are significantly higher than other varieties.

#### 3.1.3 Analysis of the quality of rice and fresh wet rice noodles from different rice varieties

Twenty-two rice varieties were analyzed for the main indices of rice quality and fresh wet rice noodle quality. The relevant indicators of rice quality and fresh wet rice noodles quality of these varieties have a wide range of changes (**Table 3**). The coefficient of variation for gel consistency was the largest among the rice quality indices at 32.66%. The average was 52.12mm, the standard deviation (SD) was 17.02, and the variation range was 22.67-79.67mm. The coefficient of variation for amylose content was the next highest at 27.28% and the variation range was 9.56-35%. The coefficient of variation for the broken noodle rate was the largest among the fresh wet rice noodles at 61.97%. The average was 17.95%, the standard deviation (SD) was 11.13, and the variation range was 3.33-43.33%. The coefficient of variation for adhesiveness was the next highest at 48.43%, with a variation range of 79.63-686.68g·s. However, the coefficient of variation for rice noodle elasticity was the smallest at 12.77%. The average was 0.62, the standard deviation (SD) was 0.08, and the variation range was 0.49-0.81. Additionally, among the main quality indices for fresh wet rice noodles, the coefficients of variation for the broken noodle rate, adhesiveness, chewiness, and spit pulp value all exceeded 30%.

**Table 3:** Statistical analysis of the quality of rice and fresh wet rice noodles from 22 rice varieties.

Type	Index	Variation range	Average	SD	CV (%)
Rice quality	Brown rice rate (%)	70.87~82.07	78.35	2.27	2.89
	Milled rice rate (%)	47.07~70.99	65.31	4.85	7.43
	Amylose content (%)	9.56~35	22.8	6.22	27.28
	Gel consistency (mm)	22.67~79.67	52.12	17.02	32.66
Fresh wet rice noodles quality	Broken noodles rate (%)	3.33~43.33	17.95	11.13	61.97
	Spit pulp value (%)	3.19~20.04	12.07	4.62	38.25
	Rice-noodles hardness (g)	2867.7~7163.87	4756.97	1175.59	24.71
	Adhesiveness (g·s)	79.63~686.68	412.42	199.73	48.43
	Rice-noodles elasticity	0.49~0.81	0.62	0.08	12.77
	Rice-noodles chewiness	885.8~4325.67	2393.07	1057.29	44.18
	Rice-noodles smell	12.8~18.8	15.78	1.8	11.39
	Rice-noodles appearance	15.2~29.2	23.53	4.06	17.26
	Texture characteristics	18~31.2	25.34	3.64	14.37
	Total score of sensory evaluation	46.8~79.2	64.61	8.65	13.38

#### 3.1.4 Correlation analysis of the quality of rice and fresh wet rice noodles from different rice varieties

A correlation analysis of the quality of rice and fresh wet rice noodles from 22 rice varieties was conducted (**Table 4**). Among the main indicators of rice quality, there was a highly significant negative correlation between brown rice rate and milled rice rate (r = 0.88**, P< 0.01), and there were highly significant correlations between all individual indices of fresh wet rice noodle quality. This suggests that the quality of rice and fresh wet rice noodles is a complex composite trait, especially among the individual indices of fresh wet rice noodles. That is, these significant or highly significant correlations lead to an overlap of the information provided by the individual indices. Therefore, it is difficult to make an accurate decision regarding the characteristics of a variety using only a single index. It is necessary to utilize multivariate analysis methods to make further analyses based on multiple indices.

**Table 4:** Correlation matrix of the main indices of rice and fresh wet rice noodles from 22 rice varieties.

Index	X1	X2	X3	X4	X5	X6	X7	X8	X9	X10	X11	X12	X13	X14
X1	1	0.88**	-0.19	-0.20	0.30	0.20	-0.12	0.13	-0.33	-0.01	-0.14	-0.29	-0.30	-0.29
X2			-0.12	-0.27	0.36	0.15	-0.06	-0.06	-0.29	0.07	-0.04	-0.28	-0.19	-0.22
X3			1	-0.04	0.10	-0.15	0.04	-0.21	0.08	0.02	0.35	0.11	0.20	0.21
X4				1	0.24	0.51*	-0.50*	0.63**	-0.40	-0.54**	-0.65**	-0.35	-0.40	-0.46*
X5					1	0.85**	-0.84**	0.66**	-0.75**	-0.79**	-0.51*	-0.84**	-0.80**	-0.83**
X6						1	-0.92**	0.82**	-0.82**	-0.86**	-0.78**	-0.87**	-0.88**	-0.94**
X7							1	-0.83**	0.89**	0.93**	0.76**	0.83**	0.86**	0.91**
X8								1	-0.75**	-0.88**	-0.83**	-0.67**	-0.85**	-0.84**
X9									1	0.82**	0.76**	0.79**	0.75**	0.84**
X10										1	0.77**	0.74**	0.84**	0.86**
X11											1	0.64**	0.77**	0.83**
X12												1	0.78**	0.93**
X13													1	0.94**
X14														1

** Significant at the 0.01 probability level; * significant at the 0.05 probability level. X1: brown rice rate; X2: milled rice rate; X3: amylose content; X4: gel consistency; X5: broken noodle rate; X6: spit pulp value; X7: hardness; X8: adhesiveness; X9: elasticity; X10: chewiness; X11: smell; X12: appearance; X13: texture characteristics; X14: total sensory evaluation score.

### 3.2 Fresh wet rice noodle raw material comprehensive evaluation model

#### 3.2.1 Solving principal components from the correlation matrix

Principal component analysis was performed for the 22 rice varieties to obtain an ANOVA contribution analysis table and component matrix for 14 indices (**Table 5**). Four principal components were extracted with a cumulative contribution of 91.442%. This is highly informative and representative and can be used to analyze 14 indices for different rice varieties.

**Table 5:** Interpretation of total variance of rice and fresh wet rice noodles quality indices of 22 rice varieties.

Principal component	Initial eigenvalue			Extract the sum of the squares of the loads		
	Total	Percentage of variance	Accumulation (%)	Total	Percentage of variance	Accumulation (%)
1	8.741	62.436	62.436	8.741	62.436	62.436
2	2.217	15.836	78.272	2.217	15.836	78.272
3	1.223	8.739	87.011	1.223	8.739	87.011
4	0.62	4.431	91.442	0.62	4.431	91.442
5	0.338	2.413	93.855			
6	0.287	2.053	95.908			
7	0.177	1.268	97.175			
8	0.146	1.046	98.222			
9	0.088	0.629	98.85			
10	0.066	0.474	99.324			
11	0.052	0.373	99.698			
12	0.033	0.236	99.934			
13	0.009	0.066	99.999			
14	0.000	0.001	100			

#### 3.2.2 Calculating principal component coefficients

The component matrix in **Table 6** shows that the main factors determining the size of composite index F1 are the 10 indices of broken noodle rate, spit pulp value, rice noodle hardness, adhesiveness, elasticity, chewiness, smell, appearance, texture characteristics, and total sensory evaluation score. This covers fresh wet rice noodle cooking quality, texture determination, and sensory evaluation. Composite index F1 is equivalent to the effect of 8.741 raw indices and corresponds to 62.436% of the raw data information. The main factors determining the size of composite index F2 are the two indices of brown rice rate and milled rice rate. This represents the appearance of rice. Composite index F2 is equivalent to the effect of 2.217 raw indices and corresponds to 15.836% of the raw data information. The main factor determining the size of composite index F3 is amylose content. This represents the cooking quality of rice. Composite index F3 is equivalent to the effect of 1.223 raw indices and corresponds to 8.739% of the raw data information. The main factor determining the size of composite index F4 is gel consistency. This also represents the cooking quality of rice. Composite index F4 is equivalent to the effect of 0.62 raw indices and corresponds to 4.431% of the raw data information. The four principal component matrices and weights were synthesized to obtain the total score coefficients for the 14 indices. From these coefficients, it can be seen that gel consistency, broken noodle rate, spit pulp value, and adhesiveness are all negative indices for the quality evaluation of fresh wet rice noodles, with the remainder being positive indices.

**Table 6:** Component matrices and weights of principal components of rice and fresh wet rice noodle quality of 22 rice varieties.

Index	Component matrices				Component weights				Total score coefficients
	F1	F2	F3	F4	F1	F2	F3	F4	F
X1	-0.26	0.89	-0.13	0.26	-0.03	0.40	-0.10	0.41	0.06
X2	-0.18	0.94	0.00	0.20	-0.02	0.42	0.00	0.33	0.08
X3	0.16	-0.15	0.91	0.30	0.02	-0.07	0.74	0.49	0.1
X4	-0.54	-0.55	-0.18	0.55	-0.06	-0.25	-0.15	0.89	-0.06
X5	-0.85	0.21	0.38	-0.14	-0.10	0.09	0.31	-0.22	-0.03
X6	-0.96	-0.02	0.03	-0.07	-0.11	-0.01	0.03	-0.11	-0.08
X7	0.95	0.11	-0.14	0.07	0.11	0.05	-0.12	0.12	0.08
X8	-0.90	-0.22	-0.15	0.07	-0.10	-0.10	-0.12	0.12	-0.09
X9	0.90	-0.12	-0.07	-0.09	0.10	-0.05	-0.06	-0.15	0.05
X10	0.92	0.24	-0.13	0.06	0.11	0.11	-0.10	0.10	0.09
X11	0.85	0.17	0.35	-0.17	0.10	0.07	0.28	-0.27	0.09
X12	0.88	-0.15	-0.12	0.15	0.10	-0.07	-0.10	0.24	0.06
X13	0.93	-0.06	0.04	0.12	0.11	-0.03	0.03	0.20	0.08
X14	0.98	-0.07	0.03	0.09	0.11	-0.03	0.02	0.14	0.08

X1, brown rice rate; X2, milled rice rate; X3, amylose content; X4, gel consistency; X5, broken noodle rate; X6, spit pulp value; X7, hardness; X8, adhesiveness; X9, elasticity; X10, chewiness; X11, smell; X12, appearance; X13, texture characteristics; X14, total sensory evaluation score.

#### 3.2.3 Principal component scores and composite scores

The linear model of four principal components with 14 indices was built from (Equation 1) **Table 5**. Taking the variance contributions of the above selected principal components 1 to 4 as weights, the principal component composite model could be calculated (Equations 2, 3). **Table 7** shows the principal component scores calculated from the principal component equations and the composite scores calculated using the ratio of the variance contribution of each principal component to the total variance contribution of the four principal components as weights. The higher scores for principal component Y1 were obtained for Zhuliangyou 4026, Yu liangyou 22, Xiangzaoxian 32, and Xiangzaoxian 24. This means that these four varieties performed better than the other eighteen varieties in terms of fresh wet rice noodle cooking quality, texture determination, and sensory evaluation. Thirteen varieties—Lingliangyou 268, Lingliangyou 942, Lingliangyou 102, Liangyouzao 17, Xinrongyou 123, Xiangzaoxian 42, Longliangyou 018, C-liangyou 343, Huailiangyou 608, Longliangyou 750, Shenliangyou 5183, T-liangyou 817, and Liangyou 336—scored negative values for principal component Y1. This shows that these thirteen varieties performed poorly for all fresh wet rice noodle indices. For principal component Y2, higher scores were obtained for C-liangyou 343 and Shenliangyou 5183. This means that these two varieties performed better than the other twenty varieties in terms of rice appearance. Seven varieties, namely, Tanliangyou 83, Lingliangyou 268, Lingliangyou 102, Liangyouzao 17, Xinrongyou 123, Liangyou 347, and T-liangyou 817, scored negative values for principal component Y2. This shows that these seven varieties performed poorly for the index of rice appearance. For principal component Y3, higher scores were obtained for Xiangliangyou No.2, Longliangyou 018, and Liangyou 336. This means that these three varieties performed better than the other nineteen varieties in terms of amylose content. Eleven varieties, Zhuliangyou 4026, Lingliangyou 268, Lingliangyou 942, Lingliangyou 102, Yuliangyou 22, Xinrongyou 123, Xiangzaoxian 42, Liangyou 5836, C-liangyou 343, Huailiangyou 608, and Shenliangyou 5183, scored negative values for principal component Y3. This shows that these eleven varieties performed poorly for the index of amylose content. For principal component Y4, higher scores were obtained for Lingliangyou 942 and Huailiangyou 608. This means that these three varieties performed better than the other twenty varieties in terms of gel consistency. Nine varieties, i.e., Zhuliangyou 4026, Lingliangyou 268, Liangyouzao 17, Yuliangyou 22, Xiangzaoxian 24, Longliangyou 018, Liangyou 5836, Longliangyou 750, and Shenliangyou 5183, scored negative values for principal component Y4. This shows that these nine varieties performed poorly for the index of gel consistency.

**Table 7:** Principal component scores and composite scores for 22 varieties of rice.

Varieties	Principal component score				Composite score	Sort
	Y1	Y2	Y3	Y4	Y	
Tanliangyou 83	0.38	-0.03	0.32	0.8	0.30	9
Zhuliangyou 4026	1.8	0.84	-0.39	-0.86	1.19	2
Lingliangyou 268	-1.05	-0.87	-2.03	-0.38	-0.99	21
Liangliangyou 942	-0.47	0.01	-0.08	1.03	-0.25	13
Lingliangyou 102	-0.3	-0.86	-0.71	0.88	-0.35	16
Liangyouzao 17	-0.02	-3.45	0.34	-1.32	-0.59	18
Zhongzao 39	0.53	0.09	0.6	0.4	0.42	8
Yuliangyou 22	1.82	0.49	-0.19	-0.34	1.19	1
Xinrongyou 123	-1.04	-0.08	-0.97	0.41	-0.73	20
Xiangzaoxian 42	-0.07	0.2	-2.06	0.62	-0.16	10
Xiangzaoxian 32	1.09	0.55	0.03	0.05	0.78	4
Xiangzaoxian 24	1.08	0.96	0.24	-0.76	0.82	3
Liangyou 347	0.81	-1.13	0.9	0.56	0.43	7
Xiangliangyou No.2	0.52	0.21	1.05	0.77	0.49	6
Longliangyou 018	-1.22	0.09	1.39	-1.06	-0.67	19
Liangyou 5836	1.12	0.63	-0.15	-0.73	0.75	5
C-liangyou 343	-0.69	1.15	-0.72	0.81	-0.28	15
Huailiangyou 608	-0.5	0.03	-0.18	1.81	-0.25	12
Longliangyou 750	-1.66	0.22	0.33	-2.28	-1.08	22
Shenliangyou 5183	-0.56	1.15	-0.63	-1.39	-0.28	14
T-you 817	-0.33	-0.27	0.67	0.01	-0.19	11
Liangyou 336	-1.26	0.07	2.23	0.97	-0.54	17

Zhuliangyou 4026 and Yuliangyou 22 achieved comprehensive scores greater than 1, significantly higher than the remaining 20 varieties. This demonstrates that the comprehensive quality of rice and fresh wet rice noodles of these two varieties was excellent. However, the overall score for Long Liangyou 750 was only -1.05. This was the smallest value among the 22 varieties, indicating that this variety performed the worst overall in terms of the quality of the rice and fresh wet rice noodles.

#### 3.2.4 Affiliation function analysis for rice and fresh wet rice noodles quality evaluation

The standardized data of the four principal components extracted from the principal component analysis were used as the base values. Calculate the value of the affiliation function of each variety in each principal component. The weights were calculated based on the ratio of the contribution of each principal component factor to the total contribution. The weights of principal components 1 to 4 were 0.6828, 0.1732, 0.0956 and 0.0485, respectively. Calculate the D-value of the composite evaluation index for the quality of different varieties of rice and fresh wet rice noodles. Varieties were ranked comprehensively based on the size of the D-value. The higher the D-value, the better the overall performance of the variety (Equation 4). As shown in **Table 8**, Yuliangyou 22 had the largest D-value of 0.88. It means that this variety performed better in the quality of rice and fresh wet rice flour. Followed by Zhuliangyou 4026. However, Longliangyou 750 had the smallest D-value of 0.20. This means that this variety showed the worst performance in rice and fresh wet rice noodles quality.

**Table 8:** 22 varieties affiliation function values, weights, D-values.

Varieties	Composite index value				Affiliation function value				D-value	Sort
	X1	X2	X3	X4	U1	U2	U3	U4		
Tanliangyou 83	0.38	-0.14	0.32	0.80	0.59	0.70	0.56	0.75	0.61	9
Zhuliangyou 4026	1.80	0.59	-0.39	-0.86	1.00	0.86	0.39	0.35	0.88	2
Lingliangyou 268	-1.05	-0.87	-2.03	-0.38	0.18	0.55	0.01	0.47	0.24	21
Liangliangyou 942	-0.47	-0.04	-0.08	1.03	0.34	0.72	0.46	0.81	0.44	13
Lingliangyou 102	-0.30	-0.88	-0.71	0.88	0.39	0.55	0.32	0.77	0.43	15
Liangyouzao 17	-0.02	-3.55	0.34	-1.31	0.47	0.00	0.56	0.24	0.39	17
Zhongzao 39	0.53	-0.04	0.60	0.40	0.63	0.72	0.62	0.66	0.65	8
Yuliangyou 22	1.82	0.27	-0.19	-0.34	1.00	0.79	0.44	0.47	0.88	1
Xinrongyou 123	-1.04	-0.05	-0.97	0.41	0.18	0.72	0.25	0.66	0.30	20
Xiangzaoxian 42	-0.07	0.12	-2.06	0.62	0.46	0.76	0.00	0.71	0.48	10
Xiangzaoxian 32	1.09	0.42	0.03	0.05	0.79	0.82	0.49	0.57	0.76	4
Xiangzaoxian 24	1.08	0.80	0.24	-0.76	0.79	0.90	0.54	0.37	0.76	3
Liangyou 347	0.81	-1.21	0.90	0.56	0.71	0.48	0.69	0.70	0.67	6
Xiangliangyou No.2	0.52	0.16	1.05	0.77	0.63	0.77	0.73	0.75	0.67	7
Longliangyou 018	-1.22	0.41	1.39	-1.06	0.13	0.82	0.80	0.30	0.32	19
Liangyou 5836	1.12	0.44	-0.15	-0.73	0.80	0.82	0.45	0.38	0.75	5
C-liangyou 343	-0.69	1.18	-0.72	0.81	0.28	0.98	0.31	0.76	0.43	16
Huailiangyou 608	-0.50	0.09	-0.18	1.80	0.33	0.75	0.44	1.00	0.45	12
Longliangyou 750	-1.66	0.62	0.33	-2.28	0.00	0.86	0.56	0.00	0.20	22
Shenliangyou 5183	-0.56	1.29	-0.62	-1.39	0.32	1.00	0.33	0.22	0.43	14
T-you 817	-0.33	-0.12	0.67	0.02	0.38	0.71	0.64	0.56	0.47	11
Liangyou 336	-1.26	0.50	2.23	0.97	0.12	0.84	1.00	0.80	0.36	18
					0.6828	0.1732	0.0956	0.0485		

#### 3.2.5 Cluster analysis of rice varieties as raw materials for fresh wet rice noodle processing

**Figure 1:**
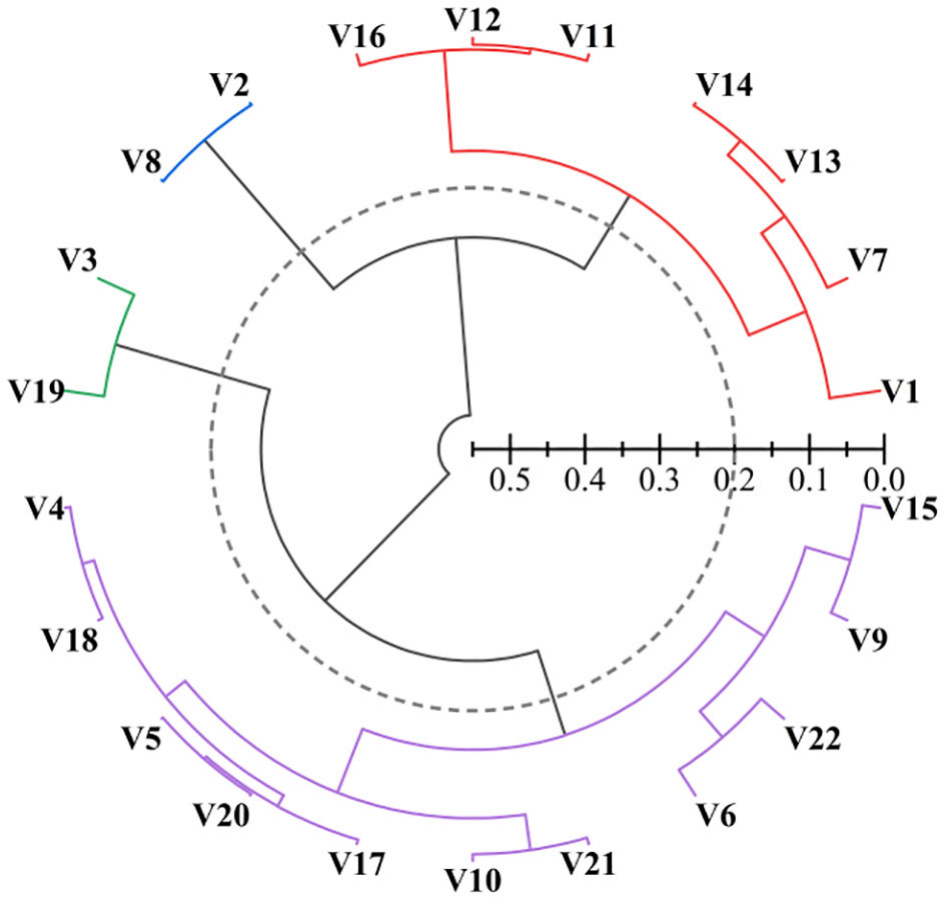
Cluster analysis of 22 rice varieties. Notes: V1: Tanliangyou 83; V2: Zhuliangyou 4026; V3: Lingliangyou 268; V4: Liangliangyou 942; V5: Lingliangyou 102; V6: Liangyouzao 17; V7: Zhongzao 39; V8: Yuliangyou 22; V9: Xinrongyou 123; V10: Xiangzaoxian 42; V11: Xiangzaoxian 32; V12: Xiangzaoxian 24; V13: Liangyou 347; V14: Xiangliangyou No.2; V15: Longliangyou 018; V16: Liangyou 5836; V17: C-liangyou 343; V18: Huailiangyou 608; V19: Longliangyou 750; V20: Shenliangyou 5183; V21: T-you 817; V22: Liangyou 336.

According to the D-values obtained through the composite evaluation of the quality of the rice and fresh wet rice noodles, twenty-two rice varieties were clustered and analyzed, and the varieties were categorized into four classes at a distance of 0.20 (**Figure 1**). The first class included Yuliangyou 22 and Zhuliangyou 4026. The noodles produced using these varieties have better comprehensive quality. The second class included Liangyou 5836, Xiangzaoxian 24, Xiangzaoxian 32, Xiangliangyou No.2, Liangyou 347, Zhongzao 39, and Tanliangyou 83. These varieties produce fresh wet rice noodles with good comprehensive quality. The third class included Longliangyou 018, Xinrongyou 123, Liangyou 336, Liangyouzao 17, T-you 817, Xiangzaoxian 42, C-Liangyou 343, Shenliangyou 5183, Lingliangyou 102, Huailiangyou 608, and Lingliangyou 942. These rice varieties produce noodles of general quality. The fourth class included Lingliangyou 268 and Longliangyou 750. These rice varieties performed poorly in terms of quality.

### 3.3 Core indicator screening and threshold verification

#### 3.3.1 Automatic linear modeling

**Figure 2:**
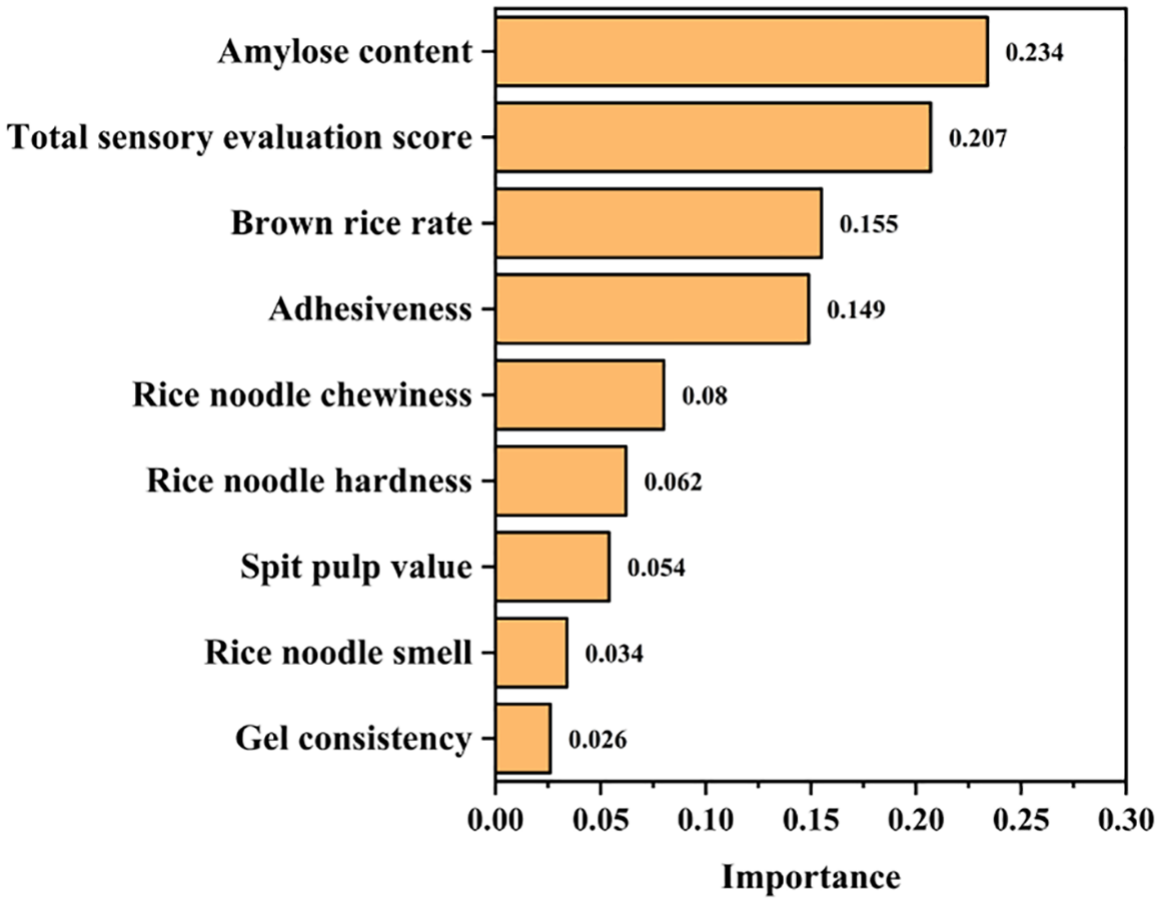
Automatic linear modeling of 14 indices.

The creation of a linear model for the fresh wet rice noodle evaluation standard was carried out using the stepwise method, with AICc as the index’s enter and remove condition. The modeling results showed that amylose content, total sensory evaluation score, brown rice rate, adhesiveness, chewiness, hardness, spit pulp value, smell, and gel consistency were selected as indices for the quick evaluation of the fresh wet rice noodle quality. Of these, the index of amylose content was the most important, reaching a value of 0.234. It is particularly interesting to see that amylose content, brown rice rate, and gel consistency, as relevant indices for rice quality evaluation, were also included in the fresh wet rice noodle quality evaluation (**Figure 2**).

#### 3.3.2 Correlation analysis of the individual index relative value and the comprehensive evaluation D-value

As shown in **Table 9**, the correlation analysis of the individual index values and the comprehensive evaluation D-value found that, in addition to the highly significant correlation between the relevant indices of fresh wet rice noodles and the comprehensive evaluation D-value, the gel consistency of rice also had a highly significant negative correlation with the comprehensive evaluation D-value (r = -0.63**, P< 0.01). It can also be used as an identification index for evaluating fresh wet rice noodle quality.

**Table 9:** Correlation of the 14 indices with comprehensive evaluation D-values.

Index	Correlation coefficient (d-value)	Significance
X1	-0.08	0.674
X2	0.01	0.994
X3	0.24	0.260
X4	-0.63	0.002
X5	-0.77	<0.0001
X6	-0.94	<0.0001
X7	0.94	<0.0001
X8	-0.93	<0.0001
X9	0.84	<0.0001
X10	0.93	<0.0001
X11	0.89	<0.0001
X12	0.83	<0.0001
X13	0.90	<0.0001
X14	0.95	<0.0001

X1: Brown rice rate; X2: Milled rice rate; X3: Amylose content; X4: Gel consistency; X5: Broken noodles rate; X6: Spit pulp value; X7: Rice-noodles hardness; X8: Adhesiveness; X9: Rice-noodles elasticity; X10: Rice-noodles chewiness; X11: Rice-noodles smell; X12: Rice-noodles appearance; X13: Texture characteristics; X14: Total score of sensory evaluation.

#### 3.3.3 Gray correlation analysis

**Figure 3:**
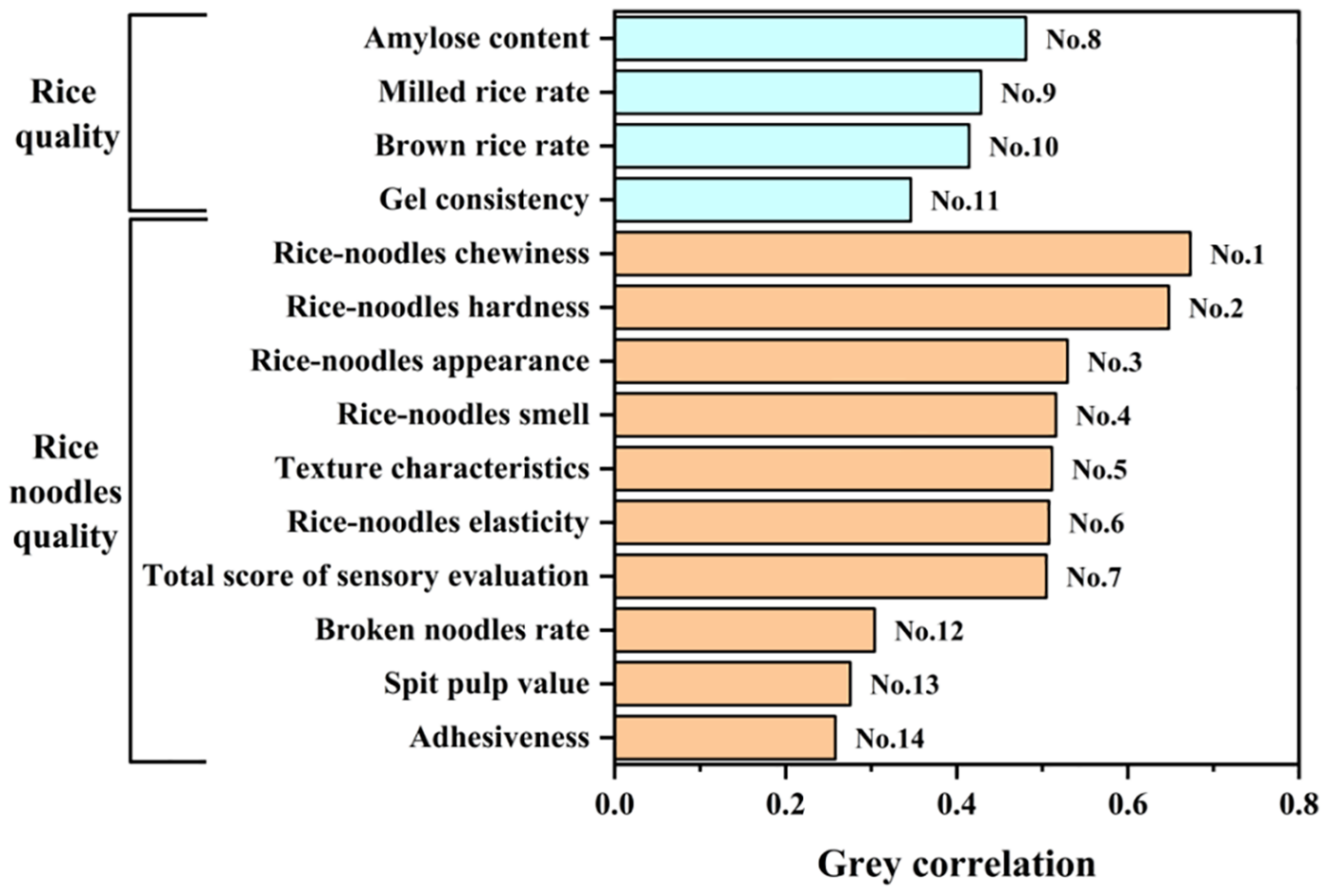
Gray correlation between the 14 indexes and the comprehensive evaluation D-value.

Gray correlation analysis showed that among the 14 indexes, rice-noodles chewiness had the highest correlation with the comprehensive evaluation D-value of 0.66635. However, adhesiveness had the lowest correlation with the comprehensive evaluation D-value of 0.26185 (Equations 5, 6). There is a large gray correlation gap between the individual indexes and the comprehensive evaluation D-value. The quality index of fresh wet rice noodles that was more closely related to the comprehensive evaluation D-value was chewiness. However, the gray correlation value of amylose with the comprehensive evaluation D-value was greater than other rice quality indexes at 0.49441 (**Figure 3**).

#### 3.3.4 Validation of the selection results of the identification indexes for fresh wet rice noodles

In order to verify the scientific validity and persuasiveness of the identification indexes, using the identification indexes obtained from any two methods mentioned above are combined as the identification indexes of the fresh wet rice noodles quality. If the real conditions allow, it can add the other indexes appropriately to support, and go to increase the precision of the fresh wet rice noodles quality identification. It is worth to note that all the multivariate analysis methods selected the close relationship between the rice quality indexes and the fresh wet rice noodles quality evaluation. Comparing the results of the clustering of amylose content, gel consistency and comprehensive evaluation D-value of 22 rice varieties, the rice varieties with the top comprehensive evaluation D-value, Yuliangyou 22, Zhuliangyou 4026, Xiangzaoxian 24, Xiangzaoxian 32, and Liangyou 5836, all satisfy the standard of the amylose content ranging between 20%-25% and the gelatin consistency less than 40mm. Among them, Xiangzaoxian 24, Xiangzaoxian 32, and Liangyou 5836 were also clustered together at 0.10 distance in the clustering based on the comprehensive evaluation D-value. That is, through the method of multivariate analysis can effectively select rice varieties for fresh wet rice noodles processing based on the combination of amylose content and gel consistency in the early stage (**Table 10**).

**Table 10:** Clustering results, D-value, amylose content and gel consistency details.

Clustering class	Varieties	D-value	Amylose content (%)	Gel consistency (mm)
Class I	Yuliangyou 22	0.88	22.25	33.00
	Zhuliangyou 4026	0.88	20.44	25.67
Class II	Xiangzaoxian 24	0.76	23.06	22.67
	Xiangzaoxian 32	0.76	24.10	36.33
	Liangyou 5836	0.75	22.12	29.67
	Liangyou 347	0.67	29.58	65.67
	Xiangliangyou No.2	0.67	29.27	58.00
	Zhongzao 39	0.65	29.69	43.33
	Tanliangyou 83	0.61	27.17	50.33
Class III	Xiangzaoxian42	0.48	10.87	68.00
	T-you 817	0.47	26.46	52.00
	Huailiangyou 608	0.45	23.80	67.67
	Lingliangyou 942	0.44	25.90	61.67
	Shenliangyou 5183	0.43	13.32	37.00
	Lingliangyou 102	0.43	20.85	75.00
	C-liangyou 343	0.43	18.59	55.33
	Liangyouzao 17	0.38	25.55	68.33
	Liangyou 336	0.36	35.00	68.33
	Longliangyou 018	0.32	26.10	45.33
	Xinrongyou 123	0.30	18.77	66.33
Class IV	Lingliangyou 268	0.24	9.56	79.67
	Longliangyou 750	0.20	19.15	37.33

Further 22 rice varieties were classified on the basis of two measures: amylose content between 20-25% and gel consistency less than 40mm. A class of varieties that satisfy a amylose content between 20-24% and gel consistency less than 40mm included Yuliangyou 22, Zhuliangyou 4026, Xiangzaoxian 24, Xiangzaoxian 32, and Liangyou 5836. The second class varieties that only satisfy the amylose content between 20-25% included Huailiangyou 608 and Lingliangyou 102. The three class varieties that only satisfy the gel consistency less than 40mm included Zhongzao 39, Shenliangyou 5183, and Longliangyou 750. The other 12 varieties of Class IV neither satisfied amylose content between 20-24% nor gel consistency less than 40mm.

**Figure 4:**
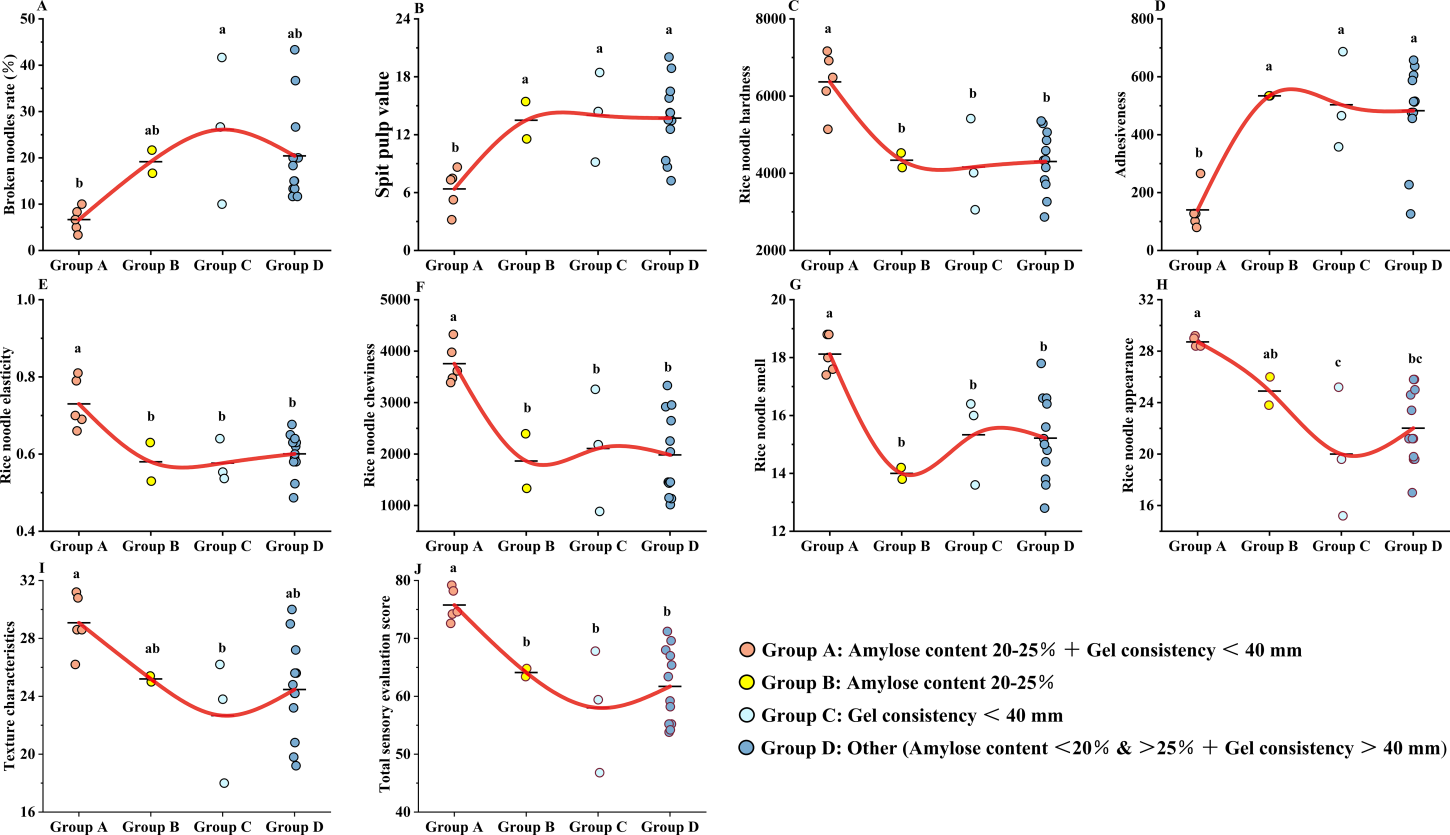
**(A)** Broken noodles rate(%)(%); **(B)** Spit pulp value(%); **(C)** Rice-noodles hardness(g); **(D)** Adhesiveness (g·s); **(E)** Rice-noodles elasticity; **(F)** Rice-noodles chewiness; **(G)** Rice-noodles smell; **(H)** Rice-noodles appearance; **(I)** Texture characteristics; **(J)** Total score of sensory evaluation.

Based on the above varietal classification, 10 indexes were trended for fresh wet rice noodles of 22 rice varieties. Broken noodles rate, spit pulp value and adhesiveness, which negatively affect the fresh wet rice noodles quality, were shown to be excellent for varieties satisfying amylose content between 20-25% and gel consistency less than 40mm. Varieties in classes II, III and IV all had higher overall trends than class I varieties. Rice-noodles hardness, rice-noodles elasticity, rice-noodles chewiness, rice-noodles smell, rice-noodles appearance, texture characteristics, and total score of sensory evaluation, which positively affect the fresh wet rice noodles quality, were shown to be excellent for varieties satisfying amylose content between 20-25% and gel consistency less than 40mm. Varieties in classes II, III and IV all had lower overall trend than class I varieties. That is, according to the selected conditions that the amylose content between 20%-25% and the gel consistency less than 40mm can be effectively selected rice varieties for fresh wet rice noodles processing (**Figure 4**).

## 4 Discussion

Fresh wet rice noodles play an important role in rice processed food, which is a large and exquisite food after rice deep processing (Li et al., 2021). Rice variety is the main factor affecting the fresh wet rice noodles quality. However, rice varieties are numerous and their quality characteristics are significantly different (Wei et al., 2022). This leads to variable quality of fresh wet rice noodles. Therefore, the quick selection of the flour rice and the establishment of its standardized evaluation system are the necessary trend of industrial production in the traditional rice noodles industry. In this study, twenty-two rice varieties were used as test materials, and 14 indexes of rice quality and fresh wet rice noodles quality were measured. Construct a comprehensive evaluation model for fresh rice raw materials through multivariate statistical methods. And screen out core indicators and thresholds.

Principal component analysis (PCA) reduces multiple correlated indicators into a few independent components and computes scores for each variety, enabling integrated evaluation (Greenacre et al., 2022). In this study, Principal component analysis was used to reduce the 14 indexes to four principal components. Based on the component matrix and principal component contribution ratio, it was further found by computing the total score coefficients of the 14 indexes that gel consistency, broken noodles rate, spit pulp value and adhesiveness played a negative role in the comprehensive evaluation of the fresh wet rice noodles quality. Luo was selected the broken noodles rate, pulp spitting value, iodine blue value and transmittance as the core indicators of the physical and chemical properties of fresh wet Rice noodles in their research results (Luo, 2012). Previous studies have also suggested selecting rice with low gel consistency when selecting raw materials for rice noodles production (Li et al., 2013). However, Jiang et al. pointed out in their research that the smaller the gel consistency, the stronger the strength, the stronger the heat resistance and the faster the gelation speed of Rice noodles gel. If the gel consistency is too small, it is easy to break the strip, and if it is too large, the powder strip is easy to bond and difficult to process (Jiang et al., 2025). Therefore, this also indicates that it is difficult to determine the criteria for the core indicator interval.

The affiliation function method is an efficient multifactor decision making method to make a comprehensive evaluation of things that are affected by multiple factors. By using the value of the affiliation function of each individual in each principal component and the ratio of the contribution of each principal component factor to the total contribution can calculate the D value of different individuals’ comprehensive evaluation index. The D-value can provide a more intuitive and convenient standard for judging the superior individuals selection. For example, by principal component analysis and affiliation function method, the rice quality of different rice varieties under different N treatments was comprehensively evaluated. Higher overall quality of rice varieties were selected (Ding et al., 2023). Using affiliation function analysis, JM418, HM19, SM22, H4399, HG35 and GY2018 were selected to have excellent drought tolerance. They can be used to research the wheat drought tolerance mechanism and breed the drought-tolerant wheat varieties (Bao et al., 2023). D-value was introduced to evaluate the low nitrogen tolerance and screened SY42, GEMS42-I and GEMS846-II. These three materials grew better and accumulated higher nitrogen under low nitrogen conditions (Miao et al., 2022). In this study, 22 rice varieties were comprehensively evaluated for rice quality and fresh wet rice noodles quality by comprehensive D-value. The results showed that fewer of the twenty-two rice varieties were evaluated with high D-values. However, most of the D-value were intermediate. This means that fewer of the twenty-two rice varieties were suitable as raw material for fresh wet rice noodles. Most of the varieties were intermediate type. Further clustering analysis revealed that twenty-two rice varieties were classified into 4 classes. Among them, Yuliangyou 22 and Zhuliangyou 4026 were in one class. These two varieties had significantly higher D-value than the other varieties.

In practical problems, a phenomenon is often associated with multiple factors. It is more efficient and practical to predict or estimate the dependent variable from many independent variables rather than from only one independent variable. The multiple linear regression method can screen the independent variables (identification indicators), leaving the useful ones and removing the useless ones. Finally, screening the best combination of independent variables together to predict or estimate the dependent variable. In this study, multiple linear regression analysis was performed by means of automated linear modeling. The results showed that amylose content, total score of sensory evaluation, brown rice rate, adhesiveness, rice-noodles chewiness, rice-noodles hardness, spit pulp value, rice-noodles smell and gel consistency were screened as indexes for quick evaluation of the fresh wet rice noodles quality. It is worth paying attention to that amylose content, brown rice rate and gel consistency were included as key indexes in the identification system for fresh wet rice noodles quality evaluation. That is, judgment on the variety’s suitability for fresh wet rice noodles can also make through the amylose content, brown rice rate and gel consistency measurements. And such results are consistent with previous studies. When the rice amylose content mass fraction is 21.0%-25.0%, the fresh wet rice noodles is soft and smooth, and the quality is the best. However, the quality of fresh rice flour made from rice with less than 21% or more than 25% of amylose content is generally poorer (Gao et al., 2015). This phenomenon may be attributed to the long molecular chains of amylose, which exhibit strong intermolecular interactions. These interactions facilitate the formation of a stable three-dimensional network structure, thereby enhancing the gel strength and stability of the starch. An amylose content exceeding 25% leads to excessively strong intermolecular forces. This results in hard and low‐viscosity cooked rice. Such properties hinder complete gelatinization during rice noodle processing. They also cause difficulty in extrusion, thereby impairing processing efficiency. Even when noodles are produced, starch granule expansion remains limited. Consequently, the rehydration rate decreases and retrogradation occurs more readily (Wang et al., 2013). In addition, Zhao et al. analyzed the synergistic effect between the fine structures of amylose and amylopectin. It was found that the different combinations and ratios of amylose and amylopectin had a significant effect on the gel consistency of rice noodles. This may also be due to the strong intermolecular and intramolecular forces that can be formed by the combination of long amylose and low amylopectin. This allows starch particles to maintain relative integrity during the extrusion process while expanding to a large extent without breaking. Therefore, it helps to increase the diameter and mechanical quality and reduce the viscosity of rice noodles (Zhang et al., 2022). As for the influence of brown rice rate on the quality of rice noodles, Xiao et al. found that the fresh wet rice noodles processed with brown rice is softer than milled rice (Xiao et al., 2021). This may be attributed to the high short-range ordered structure and relative crystallinity of brown rice starch, which also contribute to its greater hardness and chewiness, resulting in superior overall quality (Xiong et al., 2024).

In this study, further combining the correlation between comprehensive evaluation D-value and various indexes, it was found that amylose content is the rice quality index with the highest positive correlation with D-value. However, gel consistency is the rice quality index with the highest negative correlation with D-value. In the process of system development, if the trend of change of two factors is consistent. That is, the level of synchronized change is high. It means that the degree of correlation between the two is high. Conversely, it is lower. Gray correlation analysis is a method of measuring the degree of correlation between factors according to the degree of similarity or dissimilarity of trends between factors. Yi et al. used grey relational analysis to screen the indicators most closely related to low nitrogen tolerance in wheat varieties based on nitrogen fertilizer treatment, including spike yield, spike length, thousand grain weight, flag leaf length, and plant height (Zhao et al., 2021). In this study, amylose content was found as the rice quality index most closely related to D-value by gray correlation analysis. Combining the results of linear regression and correlation analysis, it was further determined that there is a close connection between amylose content, gel consistency and fresh wet rice noodles quality. They can effectively determine the fresh wet rice noodles quality and quickly screen out the flour rice varieties.

To verify that amylose content and gel consistency can be used as key indexes for quick screening of the flour rice. In this study, twenty-two rice varieties were sorted for amylose content and gel consistency. The results showed that this class of varieties with amylose content between 20%-24% and gel consistency less than 40mm shows a significant decrease of 65.22%-74.47%, 52.76%-54.46%, and 70.94%-73.74% in broken noodles rate, spit pulp value and adhesiveness compared to the class II, III, and IV varieties. While its rice-noodles hardness, rice-noodles elasticity, rice-noodles chewiness, rice-noodles smell, rice-noodles appearance, texture characteristics and total score of sensory evaluation increased significantly 46.78%-53.01%, 21.55%-26.58%, 78.15%-101.45%, 18.17%-29.43%, 15.34%-43.6%, 15.4%-28.29% and 18.19%-30.62%. Previous studies have found that too much or too little amylose content affects the rice noodles’ quality (Han et al., 2011; Yi et al., 2021; Xiao, 2022). But in general, this index doesn’t show controversy. However, gel consistency, another important factor determining the texture of rice noodles, has been controversial in the evaluation of the quality of fresh wet rice noodles (Cheng et al., 2022). In Hunan, China, the flour rice is required to have a gel consistency of less than 40 mm (Wang, 2011). But in Jiangxi, China, the gel consistency of the flour rice, Jinyou L2, reaches 74 mm (Zhong et al., 2011). In addition, Zhongjiazao 17 proved to be a suitable variety for the fresh wet rice noodles processing. Its rice noodles have a good flavor, easy to process, and fewer breaks. However, its gel consistency was 77mm (Li, 2019). In general, rice noodles with a high gel consistency are usually soft and smooth. This is suitable to make dishes such as noodle soup or stir-fried noodles. Rice noodles with a poor gel consistency have a harder texture and a looser feel. This may be more suitable for dry noodles or salads. Different regions and cultures have different preference for the gel consistency of rice noodles. Some consumers may prefer rice noodles with a soft and smooth texture, and others may prefer a firmer texture. Therefore, the gel consistency of rice noodles needs to be adjusted according to the target market and consumer preferences. The gel consistency is also related to the rice noodles’ processing characteristics. Rice noodles with a high gel consistency may require more water and more careful processing control during processing (Han et al., 2024). In addition, rice noodles with high gel consistency may be more prone to clumping or sticking during storing and transportation. Although gel consistency mainly affects the texture and taste of rice noodles. However, it may also indirectly affect the nutritional value of rice noodles. For example, rice noodles with a higher gel consistency may have a higher moisture content resulting in a lower energy density (Yang et al., 2022). The gel consistency may also affect the rice noodles’ stability and shelf life. Rice noodles with a higher gel consistency may be more easily susceptible to textural changes, such as drying, caking, or spoilage, during extended storage (Qiao et al., 2022). However, the formation of gel consistency is mainly closely related to the gelatinization process of starch. That is, under the action of heating and water, the hydrogen bond inside the starch breaks, and the structure between starch molecules is destroyed, further forming the gel structure (Gong et al., 2023). In general, the relationship between gel consistency and the fresh wet rice noodles quality is complex. It involves a number of aspects such as taste, consumer preference, processing technology, nutritional value, product stability and shelf life. Appropriate control of the gel consistency is essential to produce a high quality fresh wet rice noodles product that satisfies the market demand. Our research results mainly focus on fresh wet rice noodles. Because there are many kinds of Rice noodles. Generally, rice noodles can be divided into fresh wet rice noodles, semi dry rice noodles and dry rice noodles according to their water content. The water content of fresh wet rice noodles is 40%-65%, which is smoother and more delicate than semi dry rice noodles and dry rice noodles. Not all rice is suitable for processing into fresh wet rice noodles (Song et al., 2024; Liu et al., 2025). The evaluation criteria for each type are different.

This study primarily investigated the relationship between fresh wet rice noodle quality and the genetic characteristics of raw materials. For instance, different rice varieties vary in amylose and amylopectin content, leading to differences in noodle quality (Huang et al., 2025).Environmental factors significantly affect rice quality. High temperatures, low temperatures, insufficient or excessive light, and improper water management can alter key quality traits such as amylose content, protein content, and gel consistency (Wang et al., 2024; Zhang et al., 2025). Such variability may contribute to the difficulty in establishing uniform screening standards for rice noodle raw materials. Optimized agronomic practices can help stabilize rice quality traits, thereby mitigating the negative effects of this variability. For example, Li et al. demonstrated that improved nitrogen management increases amylose content, reduces chalkiness, and enhances the appearance quality of rice. Similarly (Li et al., 2019). Liu et al. found that controlled drainage before harvest significantly improved milling and appearance quality, as well as gel consistency and RVA profile characteristics (Liu et al., 2023). A limitation of this study is the insufficient exploration of genotype-by-management interactions. Therefore, future research should further examine the interplay between agronomic practices and genetic factors. Stabilizing rice quality performance will enhance the accuracy of screening standards for high-quality fresh wet rice noodle.

## 5 Conclusions

Fresh wet rice noodles quality is a complex trait that is influenced by many factors. In particular, there were significant correlations between the main traits of fresh wet rice noodles quality itself. By utilizing multivariate statistics, it is possible to avoid the unidirectionality of a single index and the overlap of background information of different indexes. The specific method is to show that the many indexes are transformed into several composite indexes by principal component analysis. Then on the basis of the principal component scores, the comprehensive evaluation D-value is calculated by using the affiliation function method. Based on the D-value, the fresh wet rice noodles can be judged the quality of the advantages and disadvantages. On the base of the comprehensive D-value, the cluster analysis can also provide a clear grade classification to the participating varieties. In this study, Yuliangyou 22 and Zhuliangyou 4026 were screened as high-quality rice varieties for fresh wet rice noodles processing by the above methods. By linear regression, correlation analysis and gray correlation analysis, the trait evaluation indexes for identifying the quality of fresh wet rice noodles were screened. Meanwhile, the association between rice quality and fresh wet rice noodles quality was established. That is, the two rice quality indexes of amylose content and gel consistency could effectively and quickly determine the fresh wet rice noodles characteristics. The screening conditions for the flour rice suitable for fresh wet rice noodles processing were determined to be: amylose content between 20-25% and gel consistency less than 40mm.

## Data Availability

The original contributions presented in the study are included in the article/supplementary material. Further inquiries can be directed to the corresponding authors.

## References

[B1] Amarya S. Singh K. Sabharwal M. (2015). Changes during aging and their association with malnutrition. J. Clin. Gerontology Geriatrics. 6, 78–84. doi: 10.1016/j.jcgg.2015.05.003

[B2] Bao X. Y. Hou X. Y. Duan W. W. Yin B. Z. Ren J. H. Wang Y. D.. (2023). Screening and evaluation of drought resistance traits of winter wheat in the North China plain. Front. Plant Sci. 14, 1194759. doi: 10.3389/fpls.2023.1194759, PMID: 37396647 PMC10313073

[B3] Boirie Y. Morio B. Caumon E. (2014). Cano, N. Nutrition and protein energy homeostasis in elderly. Mech. Ageing Dev. 136-137, 76–84. doi: 10.1016/j.mad.2014.01.008, PMID: 24486557

[B4] Cagampang G. B. Perez C. M. Juliano B. O. A. (1973). Gel Consistency test for eating quality of rice. J. Sci Food Agriculture. 24, 1589–1594. doi: 10.1002/jsfa.2740241214, PMID: 4771843

[B5] Cheng L. S. Liang Q. M. Yao Z. J. Lin Y. (2022). Research status and prospect of rice noodles quality evaluation and manufacture. Sci Technol. Cereals,Oils Foods. 30, 71–79. doi: 10.16210/j.cnki.1007-7561.2022.06.009

[B6] Ding M. L. Lin L. P. Li M. J. Hu X. He C. Liao H. F.. (2020). Genetic diversity analysis and comprehensive evaluation of quality traits of wheat varieties (lines) bred in Yunnan. J. South. Agriculture. 51, 255–266. doi: 10.3969/j.issn.2095-1191.2020.02.002

[B7] Ding C. Xu C. S. Lu B. Zhu X. H. Luo X. K. He B.. (2023). Comprehensive evaluation of rice qualities under different nitrogen levels in South China. Foods. 12, 697. doi: 10.3390/foods12040697, PMID: 36832772 PMC9956055

[B8] Fu X. B. (2007). Asian noodles: History, classification, raw materials, and processing. Food Res. Int. 41, 888–902. doi: 10.1016/j.foodres.2007.11.007

[B9] Gao X. X. Tong L. T. Zhong K. Liu L. Y. Zhou X. R. Zhou S. M.. (2015). Raw material selection for fresh rice noodles. J. Chin. Cereals Oils Assoc. 30, 1–5. doi: 10.20048/j.cnki.issn.1003-0174.2015.02.001

[B10] Gong X. Kou X. Y. Zhang Y. L. Li Z. Y. Tang M. R. Sun S. L.. (2023). The application of Ultra-High pressure technology in rice preservation. Packaging J. 15, 31–36 + 84. doi: 10.3969/j.issn.1674-7100.2023.03.005

[B11] Greenacre M. Groenen P. J. F. Hastie T. D’Enza A. Markos A. Tuzhilina E. (2022). Principal component analysis. Nat. Rev. Methods primers. 2, 100. doi: 10.1038/s43586-022-00184-w

[B12] Guo Y. X. Yang Z. Xu Z. M. Wang Y. Chen G. H. (2022). Different quality characterization of fresh wet rice noodles made of different early season indica rice. J. Hunan Agric. University(Natural Sciences). 48, 619–625. doi: 10.13331/j.cnki.jhau.2022.05.017

[B13] Han M. H. Cho H. J. Koh K. B. (2011). Processing properties of *Korean rice* varieties in relation to rice noodle quality. Food Sci Biotechnol. 20, 1277–1282. doi: 10.1007/s10068-011-0176-5

[B14] Han J. vid J. M. Lei D. Yang Q. Na J. Liu X.. (2024). Effects of moisture content and retrogradation on structure and properties of indica rice flour and starch gels. Food Hydrocolloids. 150, 109657. doi: 10.1016/j.foodhyd.2023.109657

[B15] Huang J. H. Xiao C. C. Zeng Y. H. Yang S. L. Wei X. Y. (2025). Amylose accumulation in grain filling stage of rice varied on amylose content. Fujian J. Agric. Sci. 40, 262–270. doi: 10.19303/j.issn.1008-0384.2025.03.006

[B16] Jiang Y. W. Xie X. Q. Zhao Y. Y. Huang L. X. Mo Q. C. Fan D. Y. (2025). The relationship between the physical and chemical properties of rice and the quality of Rice noodles and the breeding strategy of Rice noodles rice. Bull. Agric. Sci Technology. 03, 134–136. doi: 10.3969/j.issn.1000-6400.2025.03.033

[B17] Lei W. Y. Wu W. G. Liao L. Y. Ni T. Zhang Y. (2020). Quality evaluation of and raw material selection for wet rice noodle. Food Sci. 41, 74–79. doi: 10.7506/spkx1002-6630-20181202-015

[B18] Li Y. L. (2019). Exploring the characteristics and high yield cultivation techniques of Zhongjiazao 17. Agric. Dev. Equipments. 09, 194 + 196. Available online at: https:///doi.org/NJJY.0.2019-09-147 (Accessed November 14, 2019).

[B19] Li G. F. Chen. J. Lyu Y. G. Bian K. (2013). Influence of raw material properties on retrogradation characteristics of rice noodles. J. Henan Univ. Technology(Natural Sci Edition). 34, 39–42. doi: 10.16433/j.cnki.issn1673-2383.2013.02.017

[B20] Li S. X. Pu S. L. Deng F. Wang L. Hu H. Liao S.. (2019). Influence of optimized nitrogen management on the quality of medium hybrid rice under different ecological conditions. Chin. J. Eco-Agriculture 27, 1042–1052. doi: 10.13930/j.cnki.cjea.181087

[B21] Li C. M. You Y. X. Chen D. Gu Z. B. Zhang Y. Z. Holler T. P.. (2021). systematic review of rice noodles: Raw material, processing method and quality Improvement. Trends Food Sci Technology. 107, 389–400. doi: 10.1016/j.tifs.2020.11.009

[B22] Liu X. Z. Li Y. T. Xu L. C. Zheng Y. G. Bai G. Q. Li A. G. (2025). Research progress on processing and preservation technology of fresh wet rice noodles. Agric. Products Processing. 06, 88–92. doi: 10.16693/j.cnki.1671-9646(X).2025.06.019

[B23] Liu H. J. Ni X. H. Zhang L. P. Zhou J. Chen L. G. Zhang Y. F. (2023). Effect of water cut off days before harvest on rice quality of good taste rice. Jiangsu J. Agric. Sci. 39, 352–359. doi: 10.3969/j.issn.1000-4440.2023.02.007

[B24] Luo W. B. (2012). The study on the quality evaluation, processing suitability and preservation of fresh rice noodles (Changsha, Hunan: Central South University of Forestry and Technology). doi: 10.7666/d.y2097203

[B25] Luo W. B. Lin X. L. Huang L. Wu Y. Xiao H. X. Wang J. (2011). Study on physiochemical and sensory properties of fresh rice noodles produced by different varieties of indica rice. Food Machinery. 27, 7–12 + 48. doi: 10.3969/j.issn.1003-5788.2011.03.003

[B26] Miao J. J. Shi F. Li W. Zhong M. Cong L. Chen S. S. (2022). Comprehensive screening of low nitrogen tolerant maize based on multiple traits at the seedling stage. PeerJ. 10, e14218–e14218. doi: 10.e14218-e14218, PMID: 36275463 10.7717/peerj.14218 PMC9586120

[B27] Mou X. W. Song Y. F. Chen L. T. (2022). Research status and prospect of key technologies for automatic production of fresh and wet rice flour. Food Machinery. 38, 110–117. doi: 10.13652/j.spjx.1003.5788.2022.90209

[B28] P J. S. Zhu S. L. Lin X. Wan X. Zhang Q. Njie A.. (2023). Evaluation of preharvest melatonin on soft rot and quality of kiwifruit based on principal component analysis. Foods (Basel Switzerland). 12 (7). doi: 10.3390/FOODS12071414, PMID: 37048235 PMC10093534

[B29] Qiao C. C. Tian X. H. Wang L. X. Jiang P. Zhai X. T. Wu N. N.. (2022). Quality characteristics, texture properties and moisture migration of fresh brown rice noodles under different storage and temperatures conditions. J. Cereal Sci., 103434. doi: 10.1016/J.JCS.2022.103434

[B30] Shuang W. Yao X. H. Wang K. L. Yang S. P. Ren H. D. Huang M.. (2022). Quality analysis and comprehensive evaluation of fruits from different cultivars of pecan (*Carya illinoinensis* (Wangenheim) K. Koch). Forests. 13, 746–746. doi: 10.3390/F13050746

[B31] Song X. Feng W. Wang T. Zhang H. Chen Z. X. Wang,. R. (2024). Effect of different milling methods on the physicochemical properties of rice flour and the qualities of fresh rice noodles. Sci Technol. Cereals Oils Foods. 32, 42–50. doi: 10.16210/j.cnki.1007-7561.2024.05.006

[B32] Tang R. Long L. Zhu Z. Xiong N. Yu D. Liu L.. (2018). “General administration of quality supervision, inspection and quarantine of the people’s republic of China, standardization administration of the people’s republic of China,” in GB/T 17891-2017, high quality paddy (Beijing, China: China Standards Press), 1–10.

[B33] Tsukasa M. (2019). Rice Flour: A promising food material for nutrition and global health. J. Nutr. Sci Vitaminology. 65, S13–S17. doi: 10.3177/jnsv.65.S13, PMID: 31619613

[B34] Wang X. H. (2011). Study on the selection of rice noodles varieties and their cultivation techniques (Changsha, Hunan: Hunan Agricultural University). Available online at: https://kns.cnki.net/kcms2/article/abstract?v=bTgd32KJj6t6ybwbgIIQfTl3NkJg6x5XlEEDGzvOxM2kySS0Sh92DGxifH2zCOOroj3QwE0vEt6zgUBaLvQBX1eqdCXQB10fOpE45-ZC6fMZc7IXN6jQxeMNNPlx8Knq7_7wOLzho0yP5llofAwGFmUwQhspjl_smGp3tTi3HFspKYRoG6NCL9UOJC_8jJ0d&uniplatform=NZKPT&language=CHS (Accessed May 5, 2025).

[B35] Wang J. Jing X. Wang K. Wang W. Zhou M. Liu Z. Q.. (2024). Research progress on effects of high temperature and drought stresses during grain filling stage on rice quality. Shandong Agric. Sci. 56, 165–172. doi: 10.14083/j.issn.1001-4942.2024.08.024

[B36] Wang A. N. Qin J. Tang X. H. Zhao Z. G. Chen X. Dai J. J.. (2025). ruit quality analysis and comprehensive evaluation of 27 avocado varieties. J. Fruit Sci., 1–19, 1-19. doi: 10.13925/j.cnki.gsxb.20250202

[B37] Wang Y. H. Zhang Y. H. Zhang M. W. Wei Z. C. Tang X. J. Zhang R. F.. (2013). Effect of amylose content of different rice varieties on the qualities of rice vermicelli. Scientia Agricultura Sin. 46, 109–120. doi: 10.3864/j.issn.0578-1752.2013.01.013

[B38] Wei P. J. Chen J. Xu F. Chen L. (2020). Effects of indica rice varieties on the quality of instant fresh rice noodles. J. Henan Univ. Technology(Natural Sci Edition) 41 (05), 38–43+49. doi: 10.16433/j.1673-2383.2020.05.006

[B39] Wei P. Fang F. Liu G. M. Zhang Y. Y. Wei L. Y. Zhou K.. (2022). Effects of composition, thermal, and theological properties of rice raw material on rice noodle quality. Front. Nutr. 9, 1003657. doi: 10.3389/FNUT.2022.1003657, PMID: 36118753 PMC9479187

[B40] Wu M. Y. Liao L. Y. Ren X. L. Liu C. Wu W. G. (2025). Effects of semi-dry milling with different pre-drying methods on the quality of rice powder and its fresh wet rice powder. Cereals Oils 38, 37–43. doi: 10.3969/j.issn.1008-9578.2025.05.007

[B41] Xiao Z. W. (2022). Huang, M. Fresh Rice noodle Qual. influencing factors. China Rice. 28, 34–41. doi: 10.3969/j.issn.1006-8082.2022.03.006

[B42] Xiao Z. W. Chen J. N. Cao F. B. (2021). Yield and quality of brown rice noodles processed from early-season rice grains. Sci. Rep. 11, 18668–18668. doi: 10.1038/s41598-021-98352-7, PMID: 34548582 PMC8455603

[B43] Xiong S. H. Yang X. L. Chen T. T. Luo S. J. Liu C. M. (2024). The effect of brown rice flour particle size on the quality of brown rice noodles. Food Machinery 40 (09), 174–178+199. doi: 10.13652/j.spjx.1003.5788.2024.80401

[B44] Xuan Y. Yi Y. Liang H. Wei S. Q. Chen N. P. Jiang L. G.. (2020). Amylose content and RVA profile characteristics of noodle rice under different conditions. Agron. J. 112, 117–129. doi: 10.1002/agj2.20079

[B45] Yan X. Y. Wu J. Y. Zhao C. H. Luo S. J. Huang L. Guo D. B.. (2023). Chinese rice noodles form the viscoelastic texture by dual high-temperature retrogradation: An insight into the mechanism. LWT. 189, 115496. doi: 10.1016/j.lwt.2023.115496

[B46] Yang S. Jin L. Xu X. H. Shan C. S. Chen Z. G. (2022). Long-term storage and temperature induced quality changes of industrial-scale wet starch noodles. LWT 153. doi: 10.1016/j.lwt.2021.112504

[B47] Yi C. P. Zhu H. Zhang Y. Wu S. X. Bao J. S. (2021). The role of indica starch in the mechanism of formation of fresh rice noodles. J. Cereal Sci. 99. doi: 10.1016/J.JCS.2021.103212

[B48] Zhang C. M. Guo S. J. Kang S. Qi Y. Y. Zhu Z. R. Ye L. H.. (2025). Comprehensive evaluation of straw forage quality of different soybean varieties based on principal component analysis and membership function analysis. Feed Res. 48, 143–148. doi: 10.13557/j.cnki.issn1002-2813.2025.07.026

[B49] Zhang J. Y. Kong H. C. Ban X. F. Li C. M. Gu Z. B. Li Z. F. (2022). Rice Noodle quality is structurally driven by the synergistic effect between amylose chain length and amylopectin Unit-Chain ratio. Carbohydr. Polymers. 295, 119834. doi: 10.1016/j.carbpol.2022.119834, PMID: 35989031

[B50] Zhang Z. P. Liu S. Li J. Qi J. Shen J. Wang X. L.. (2025). Comprehensive exploration of impacts of ecological factors on rice quality and improvement strategie. Barley Cereal Sci. 42, 1–6 + 16. doi: 10.14069/j.cnki.32-1769/s.2025.03.001

[B51] Zhang X. Y. Wu G. F. Wu Y. H. Tang N. Huang L. Dai D. Q.. (2024). Diversity analysis and comprehensive evaluation of 101 soybean (*Glycine max L.*) germplasms based on sprout quality characteristics. Foods. 13, 3524–3524. doi: 10.3390/FOODS13213524, PMID: 39517308 PMC11545536

[B52] Zhang C. N. Xue W. Feng X. Y. Xue Z. C. Wang J. R. Chen Z. X.. (2021). Adaptability of rice noodle raw material and rice blending. J. Chin. Cereals Oils Assoc. 36, 1–7. doi: 10.3969/j.issn.1003-0174.2021.11.002

[B53] Zhao R. Zhang X. H. ZHANG C. Y. Guo J. L. Wang Y. Li H. X. (2021). Evaluation and screening of nitrogen efficiency of wheat germplasm resources at mature stage. Scientia Agricultura Sinica. 54, 3818–3833. doi: 10.3864/j.issn.0578-1752.2021.18.003

[B54] Zhong Y. Y. Liu H. S. Zhou Q. Sun M. Y. Hu L. X. Chen Z. L.. (2011). Characteristics of rice noodles variety Jinyou L2 in Jiangxi province and its high yield and high efficiency cultivation techniques. Modern Agric. Sci Technology. 18, 69–70. doi: 10.3969/j.issn.1007-5739.2011.18.044

[B55] Zhou X. Q. Peng C. Zhang Y. R. Guo L. L. Xiong N. (2018). Quality analysis of early indica rice cultivars and their suitability for processing of pressed fresh noodles. Food Sci. 39, 36–43. doi: 10.7506/spkx1002-6630-201819007

[B56] Zhu Y. D. Wang H. Q. Wang H. Z. Ren H. Lyu J. H. Zhao B.. (2022). Evaluation and identification index of heat tolerance in different summer maize varieties at V12 stage. Acta Agronomica Sinica. 48, 3130–3143. doi: 10.3724/SP.J.1006.2022.13079

[B57] Zou J. Hu W. Li Y. X. He J. Q. Zhu H. Zhou Z. G. (2020). Screening of drought resistance indices and evaluation of drought resistance in cotton (*Gossypium Hirsutum* L.). J. Integr. Agriculture. 19, 495–508. doi: 10.1016/S2095-3119(19)62696-1

